# Off-target based drug repurposing opportunities for tivantinib in acute myeloid leukemia

**DOI:** 10.1038/s41598-018-37174-6

**Published:** 2019-01-24

**Authors:** Brent M. Kuenzi, Lily L. Remsing Rix, Fumi Kinose, Jodi L. Kroeger, Jeffrey E. Lancet, Eric Padron, Uwe Rix

**Affiliations:** 1 Department of Drug Discovery, H. Lee Moffitt Cancer Center & Research Institute, Tampa, Florida 33612 United States; 2 Department of Hematologic Malignancies, H. Lee Moffitt Cancer Center & Research Institute, Tampa, Florida 33612 United States; 3 Department of Thoracic Oncology, H. Lee Moffitt Cancer Center & Research Institute, Tampa, Florida 33612 United States; 4 Flow Cytometry Core, H. Lee Moffitt Cancer Center & Research Institute, Tampa, Florida 33612 United States; 5 Cancer Biology Ph.D. Program, University of South Florida, Tampa, Florida 33620 United States

## Abstract

GSK3α has been identified as a new target in the treatment of acute myeloid leukemia (AML). However, most GSK3 inhibitors lack specificity for GSK3α over GSK3β and other kinases. We have previously shown in lung cancer cells that GSK3α and to a lesser extent GSK3β are inhibited by the advanced clinical candidate tivantinib (ARQ197), which was designed as a MET inhibitor. Thus, we hypothesized that tivantinib would be an effective therapy for the treatment of AML. Here, we show that tivantinib has potent anticancer activity across several AML cell lines and primary patient cells. Tivantinib strongly induced apoptosis, differentiation and G2/M cell cycle arrest and caused less undesirable stabilization of β-catenin compared to the pan-GSK3 inhibitor LiCl. Subsequent drug combination studies identified the BCL-2 inhibitor ABT-199 to synergize with tivantinib while cytarabine combination with tivantinib was antagonistic. Interestingly, the addition of ABT-199 to tivantinib completely abrogated tivantinib induced β-catenin stabilization. Tivantinib alone, or in combination with ABT-199, downregulated anti-apoptotic MCL-1 and BCL-XL levels, which likely contribute to the observed synergy. Importantly, tivantinib as single agent or in combination with ABT-199 significantly inhibited the colony forming capacity of primary patient AML bone marrow mononuclear cells. In summary, tivantinib is a novel GSK3α/β inhibitor that potently kills AML cells and tivantinib single agent or combination therapy with ABT-199 may represent attractive new therapeutic opportunities for AML.

## Introduction

Despite significant advances in targeted therapy development and a growing repertoire of drugs being tested in the treatment of acute myeloid leukemia (AML)^1^, patient outcomes for AML have changed little in the last several decades. Only a small percentage of genetically defined AML patients exhibit durable long-term responses with current therapy. For instance, identification of the FLT3 internal tandem duplication mutation in 13–36% of AML (depending on the subgroup)^2^ has led to the development of the FLT3 inhibitors quizartinib and midostaurin^3^, the latter of which has recently received FDA approval in combination with standard cytarabine and daunorubicin. However, the 5-year overall survival rates of the majority of AML cases ranges from 5–15% in older patients to 30% in young adults^4^. This lack of improvement in patient survival rates is primarily attributed to the limited efficacy of currently available therapies in AML and the need for new targeted drugs. Although a number of promising drug candidates are being tested, such as the above mentioned FLT3 inhibitors, combination chemotherapy remains the standard of care^3^. Thus, there persists a clear unmet need for new drugs for the treatment of AML.

Through the combination of chemical and RNAi screens, it has been suggested that GSK3α is a novel target in AML^5^. In contrast to the more established role of GSK3α/β as a tumor suppressor pair, which inhibits Wnt signaling via β-catenin phosphorylation and subsequent degradation^6^, it has been shown that GSK3α plays an important role in maintaining an undifferentiated leukemic state of AML blasts and therefore *selective* targeting of GSK3α, which avoids concomitant inhibition of GSK3β and β-catenin stabilization, could represent a viable therapeutic strategy in AML^5^. Currently, the only FDA-approved GSK3 inhibitor is lithium chloride (LiCl), which is approved for the treatment of epilepsy and bipolar disorder^7,8^. However, given the narrow therapeutic index of LiCl, the lack of GSK3α specificity, and its limited kinome-wide selectivity^9,10^, its utility as an AML therapy is questionable. There are a number of GSK3 inhibitors in development, but current compounds are either highly unselective featuring various off-targets in addition to GSK3α/β, lack isoform selectivity or have not yet advanced to clinical studies^11,12^. We have previously identified GSK3α/β as novel targets of tivantinib (ARQ197)^13^, an advanced clinical drug candidate, which was initially thought to be a highly specific MET inhibitor^14^. We observed that tivantinib, compared to other GSK3 inhibitors, has remarkable kinome-wide selectivity for GSK3α/β, as well as a slight preference for GSK3α over GSK3β. Considering the identification of GSK3α as a potential pro-tumorigenic signaling protein, we hypothesized that tivantinib may be an effective, novel therapeutic option for AML. In the current study, we therefore characterized tivantinib’s anticancer activity in AML cell lines, identified a synergistic drug combination with the BCL-2 inhibitor ABT-199, and demonstrated its efficacy in primary AML samples. The results presented herein suggest that tivantinib, either as a single agent or in combination with ABT-199, may be a novel and attractive targeted therapy option for AML.

## Materials and Methods

### Cell culture and reagents

HL60 cells were kindly provided by Dr. G. Reuther (Moffitt Cancer Center, Tampa FL) and were cultured in IMDM (20% FBS). U937 cells were a kind gift from Dr. G. Superti-Furga (CeMM, Vienna, Austria) and were cultured in RPMI 1640 (10% FBS). Cell line authentication was done by short-tandem repeat (STR) analysis. Tivantinib (Moffitt Chemistry Core and ChemieTek), ABT-199 (ChemieTek), PF-04217903 (Selleckchem) and 6-bromoindirubin-3′-oxime (BIO, Cayman Chemical) were dissolved in DMSO (10 mM) and LiCl and NaCl (Sigma-Aldrich) were dissolved in sterile diH_2_O (10 M and 6 M, respectively).

### Immunoblotting

Cells were lyzed using 0.20% NP40, 50 mM Tris pH 7.5, 5% Glycerol, 1.5 mM MgCl_2_, 100 mM NaCl lysis buffer containing Phosphatase Inhibitor Cocktail 2 (Sigma, P5726) and cOmplete Protease Inhibitor Cocktail (Roche, 11873580001). Lysates were resolved by SDS-PAGE and incubated with primary antibodies. Antibodies used were against actin (Sigma, A5441) and pGSK3 (Y279/216) (Millipore, 05-413). Antibodies against β-catenin (sc-7199), Bak (sc-832), and MCL-1 (sc-819) were from Santa Cruz. Antibodies against GSK3α (#4337), GSK3β (#9315), pSer10 Histone H3 (#3377), Cleaved Caspase 3 (#9661), PARP-1 (#9542), and BCL-XL (#2764) were from Cell Signaling. Secondary antibodies were HRP-conjugated α-rabbit or α-mouse (GE Healthcare).

### Viability assays and synergy calculations

Cell viability assays were conducted according to manufacturer’s specifications for CellTiter-Glo Luminescent Cell Viability Assay (Promega). Cells were seeded at 1000 or 3000 cells/well in a 384 well microtiter plate and treated after 24 hours with drug diluted in the respective culture medium at the indicated concentrations. Cells were treated for 72 hours before the addition of CellTiter-Glo reagent and read on a M5 Spectramax plate reader (Molecular Devices). Raw data was normalized to DMSO controls and a three-parameter log-logistic function was fit to the data for an IC_50_ estimation using R. For the synergy screen, tivantinib was plated at 0 µM and 0.25 µM, respectively. Each library drug was tested at 0.5 µM and 2.5 µM, respectively. Subsequent drug combination effects were evaluated by the Bliss method.

### Flow cytometry

Cells were treated as indicated with DMSO, NaCl, LiCl, or tivantinib. For cell cycle experiments, cells were harvested following incubation, fixed with 70% cold ethanol and stored at −20 °C until analyzed. Cells were washed with PBS and cell cycle was determined by incubating in a 1 μg/mL DAPI (4′,6-Diamidino-2-phenylindole, Sigma)/0.1% Triton X-100/PBS solution. For apoptosis experiments, treated cells were stained with Annexin V–APC (BD Biosciences) and 100 ng/mL DAPI according to the manufacturer’s instructions. PE Mouse Anti-Human CD11b/Mac-1 (BD Biosciences) was used to monitor cell differentiation. Analyses were conducted using a FACSCanto II benchtop analyzer (BD Biosciences). Cell cycle was analyzed using ModFitLT V3.2.1 (Verity Software House). Apoptosis and differentiation data was analyzed using Flowjo (Treestar, Inc.). Analyses represent data for singlet cells using a standard aggregate gating strategy.

### Colony formation assays

AML patient bone marrow mononuclear cells (BMNCs) were seeded into 6-well plates and treated overnight at the indicated concentrations of drug using IMDM (10% FBS) as the diluent. Treated cells were then collected and suspended in MethoCult™ H4034 Optimum methylcellulose medium (StemCell Technologies) containing additional drug, split into technical duplicates (200,000 cells/replicate) and plated in 30 mm cell culture dishes. A colony was defined as a cell cluster containing >30 cells. Colonies were counted manually following 14 days of growth. Select samples were chosen for an additional readout after 19 days. Average colonies and standard deviation were calculated for each treatment. Samples for this project were archived and retrieved under both SRC and IRB approval for the Total Cancer Care® and Moffitt Cancer Center pilot protocol.

### Gene expression profiling data analysis

GSK3α and GSK3β expression levels across different human myeloid lineages were queried using the Bloodpool aggregation of hematopoietic expression profiles from numerous studies catalogued in the manually curated BloodSpot database^15^, which provides gene expression profiles of a number of mouse/human hematopoietic cells (normal and AML).

### Gene silencing sensitivity profiling data analysis

shGSK3α and shGSK3β sensitivity data was downloaded from the Project DRIVE database (https://oncologynibr.shinyapps.io/drive/)^16^ and imported into python for analysis. The redundant siRNA activity (RSA) score was used as the sensitivity measurement as the RSA sensitivity is calculated using all shRNA reagents against a given gene to determine a score^17^.

### Tivantinib sensitivity prediction

In order to predict sensitivity of AML cell lines to tivantinib, we built a regularized linear regression model (elastic net) to select gene features that can predict a tivantinib response vector. Elastic net regularization is a machine learning algorithm that is specially suited for the case of many more input features (genes) than samples (cell lines). Candidate predictive features were selected from 18989 genes with normalized measures of gene expression in CCLE for cell lines that have tivantinib sensitivity data in CTRPv2 (n = 297)^18^. Data was split into training (0.75) and test sets (0.25). Let $X\in {{\mathbb{R}}}^{nxp}$ be the matrix of predictive features, where *n* is the number of cell lines included in the training set and *p* is the number of features. Let $y\in {{\mathbb{R}}}^{n}$ be the vector of sensitivity values for the same cell line panel. The elastic net attempts to find the weighted (β) linear combination of columns of features (genes) that can best approximate tivantinib AUC (*y*) or by solving the following:

$$argmi{n}_{\beta }\{||y-X\beta |{|}_{2}^{2}+\lambda (\alpha ||\beta |{|}_{2}^{2}+(1-a)||\beta |{|}_{1})\}$$

where λ and α are tunable parameters where λ controls the overall penalty and α controls the mixing ratio of L1- and L2-norm. We optimized λ and α for the model with a tuning grid of 1000 values of λ from 10e-10 to 10e10 and 10 values of α from 0 to 1 using 10000 iterations of 10-fold cross validation. The values of λ and α were chosen to be those that minimized the root mean square error for each fold. The trained model was then used to predict AML cell line sensitivity (n = 34) to tivantinib (for which data does not exist in CTRPv2 and thus not used in model training). Statistically significant differences in tivantinib sensitivity between AML and non-AML cell lines was determined by Kolmogorov-Smirnov test.

### Proteomics and bioinformatic analysis

Drug affinity chromatography experiments were conducted essentially as described previously^13^. Briefly, c-(−)-tivantinib, c-(+)-tivantinib, and ampicillin were immobilized on NHS-activated Sepharose for Fast Flow resin (GE Healthcare). Non-coupled resin (blocked beads) and coupled resin were then blocked with ethanolamine for 4 hours. HL60 and U937 cells were lyzed and total cell lysate containing 10 mg of protein were added to the affinity matrix for 6 hours. Competition experiments were conducted by incubating total cell lysates with 20 μM BIO for 2 hours prior to affinity chromatography. Blocked beads were incubated with lysate without immobilized compound.

A nanoflow ultra high performance liquid chromatograph (RSLC, Dionex) coupled to an electrospray bench top orbitrap mass spectrometer (Q-Exactive plus, Thermo Fisher) was used for tandem mass spectrometry peptide sequencing experiments. Samples were first loaded onto a pre-column (2 cm × 100 µm ID packed with C18 reversed-phase resin, 5 µm, 100 Å) and washed for 8 minutes with aqueous 2% acetonitrile (ACN) and 0.04% trifluoroacetic acid. The trapped peptides were eluted onto the analytical column, (C18, 75 µm ID × 50 cm, 2 µm, 100 Å, Dionex). The 120-minute gradient was programmed as: 95% solvent A (2% ACN + 0.1% formic acid) for 8 minutes, solvent B (90% ACN + 0.1% formic acid) from 5% to 50% in 90 minutes, then solvent B from 50% to 90% B in 7 minutes and held at 90% for 5 minutes, followed by solvent B from 90% to 5% in 1 minute and re-equilibrate for 10 minutes. The flow rate on the analytical column was 300 nl/min. Sixteen tandem mass spectra were collected in a data-dependent manner following each survey scan. Both MS and MS/MS scans were performed in the Orbitrap to obtain accurate mass measurements using 60 second exclusion for previously sampled peptide peaks. Mascot searches were performed against the Swiss-Prot human database downloaded on June 12, 2014^19^. Two trypsin missed cleavages were allowed, the precursor mass tolerance was 10 ppm. MS/MS mass tolerance was 0.05 Da. Dynamic modifications included carbamidomethylation (Cys) and oxidation (Met). Mascot search results were summarized in Scaffold 4.3.

Subsequently data was imported into Galaxy for analysis with APOSTL^20,21^. Data was formatted into inter, prey and bait files using total spectral counts as a measure of abundance and ampicillin drug affinity chromatography experiments as negative controls. Data was then analyzed by SAINTexpress and the CRAPome within APOSTL to determine the probability of selective interactions of proteins with tivantinib^22,23^. The resulting files were merged and summarized in APOSTL’s interactive environment for analysis and visualization (Table S1).

## Results

### GSK3α is a drug target in AML cells

GSK3α has been described to be a novel target in AML^5^. Supporting this report, analysis of publically available expression levels of GSK3α and GSK3β using the BloodSpot database (which contains more than 2000 AML and normal samples assembled from six independent studies on AML) revealed that GSK3α is overexpressed across multiple AML subtypes as compared to normal hematopoietic lineages (Fig. S1a). Interestingly, GSK3β expression in AML differs little from normal hematopoiesis (Fig. S1b). In order to evaluate AML sensitivity to GSK3α/β gene silencing, we analyzed the publically available shRNA screening data in Project DRIVE^16^, which contains the cell viability data following shRNA gene silencing of various genes across 384 cancer cell lines. Consistent with GSK3α being overexpressed in AML, this analysis suggested that AML cell lines are significantly more sensitive (low RSA Sensitivity score) to GSK3α silencing as compared to GSK3β gene silencing (Fig. S1c). Furthermore, AML cell lines constitute the most sensitive population of hematopoietic cell lines with regard to GSK3α gene silencing (Fig. S1d). Taken together these analyses support that GSK3α is an actionable target in AML cell lines.

### Tivantinib potently inhibits viability of AML cells

**Figure 1:**
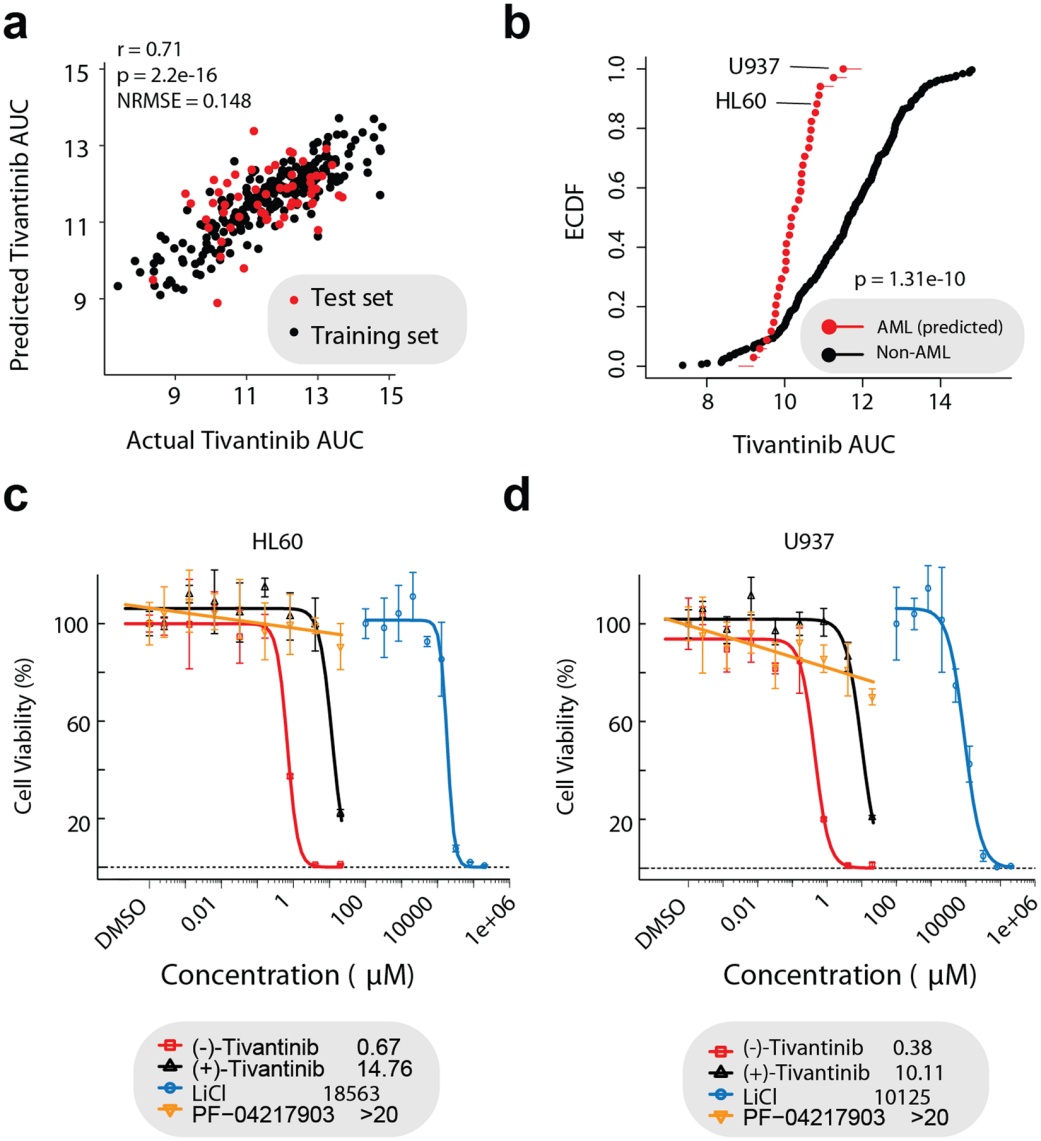
Effects of tivantinib on AML cell viability. (**a**) Correlation of predicted vs. actual area under the curve (AUC) values across all cell lines in the training and test sets. NRMSE = Normalized Root Mean Square Error. (**b**) Empirical cumulative distribution function (ECDF) comparing the predicted AML AUC values to all the non-AML AUC values in CTRPv2. Statistical significance was determined using a Kolmogorov-Smirnov test. (**c–d**) Dose response curves and IC_50_ values for inhibition of viability by (−)-tivantinib, (+)-tivantinib, LiCl and PF-04217903 of (**c**) HL60 and (**d**) U937 cells following 72 h treatment. Displayed concentrations are in μM.

Since we had previously identified GSK3α as a prominent tivantinib target^13^, we wanted to determine tivantinib’s efficacy in AML cells. To the best of our knowledge, tivantinib has never been tested across multiple AML cell lines, including the various large drug screening efforts such as the Cancer Therapeutic Response Portal v2 (CTRPv2)^18^. Therefore, to evaluate tivantinib’s efficacy across all AML cell lines in the Cancer Cell Line Encyclopedia (CCLE)^24^, we trained an elastic net regularized regression model to predict the area under the curve (AUC) sensitivity values of all cell lines with tivantinib sensitivity data in CTRPv2 (which does not include tivantinib sensitivity information for AML cell lines) using the gene expression profiles of these cell lines (CCLE) as features. Our model had good accordance between predicted and experimental values (r = 0.71, AUC = 0.83) which is comparable to similar models (Figs 1a, S2a)^25^. Interestingly, many of the gene features selected through regularization are known to associate with GSK3 signaling (STRING)^26^, such as the TCF7 cofactor MLLT11, which had the most highly weighted coefficient (Fig. S2b)^27^. We then applied this model to all AML cell lines in CCLE using their gene expression profiles, which predicted AML cell lines to be sensitive to tivantinib treatment. Interestingly, AML cell lines were predicted to be significantly more sensitive to tivantinib than non-AML cell lines (Fig. 1b).

In order to validate the predicted AML sensitivity to tivantinib, we treated HL60 and U937 AML cell lines with (−)-tivantinib, which is currently in advanced clinical development, its enantiomer (+)-tivantinib, which is a much weaker GSK3 inhibitor, the *bona fide* pan-GSK3α/β inhibitor LiCl and the MET inhibitor PF-04217903 as indicated. Intriguingly (−)-tivantinib, but not (+)-tivantinib, displayed nanomolar efficacy in HL60 and U937 cell lines (Fig. 1c,d). This is in accordance with our previous results in MLL-rearranged acute lymphoblastic leukemia (ALL) cell lines, which are known to be sensitive to GSK3 inhibition^13,28^. As expected, LiCl also showed strong activity (10–20 mM is a widely used concentration relevant for GSK3 inhibition by LiCl^5^, which as a salt has a different mechanism of action) while the potent and selective MET-inhibitor PF-04217903 was essentially inactive suggesting that GSK3, not MET, inhibition is responsible for tivantinib’s activity in AML cells (Fig. 1c,d). Since tivantinib has previously been suggested to elicit anticancer activity in NSCLC through disruption of microtubule dynamics^29,30^, we further evaluated the relative contribution that inhibition of MET, GSK3 or microtubule polymerization plays in tivantinib’s mechanism of action in these cells. We trained additional elastic net regularized regression models to predict paclitaxel (microtubule inhibitor), SGX253 (MET inhibitor), and ML320 (highly selective GSK3 inhibitor) sensitivity across cell lines present in CTRPv2. We then applied these models to predict AML sensitivity, and performed pairwise comparisons (Spearman) of the model predictions. As expected, tivantinib’s sensitivity profile was uncorrelated with SGX253 further supporting that MET is not involved in tivantinib’s mechanism in these cells. Interestingly, whereas tivantinib was only weakly correlated with paclitaxel, it was highly correlated with ML320 suggesting the GSK3 inhibition is the primary mechanism in which tivantinib elicits activity in these cells (Fig. S2c). In summary, this data demonstrates that tivantinib harbors potent anticancer activity in AML cell lines and this activity can likely be explained by GSK3 inhibition.

### Tivantinib binds GSK3α/β in AML cells

**Figure 2:**
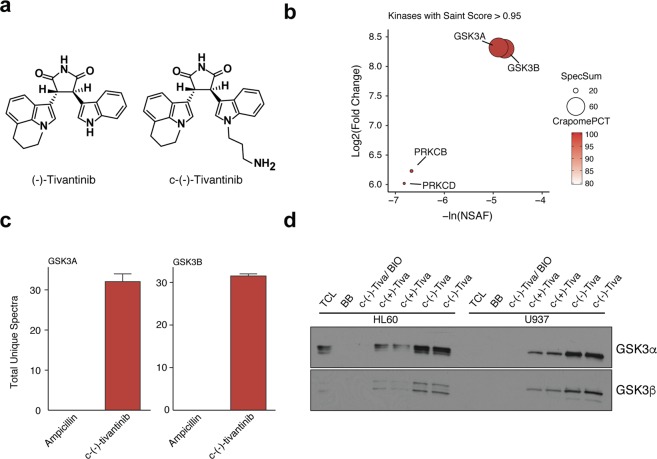
Proteomic analysis of tivantinib’s target profile. (**a**) Chemical structures of (−)-tivantinib and couplable c-(−)-tivantinib (**b**) Kinases enriched from drug affinity chromatography in HL60 cells passing SaintScore >0.95, CRAPomePCT ≥ 95%, and -ln(NSAF) ≥ −7 cutoffs. Bubble size represents the sum of total unique spectra. Bubble color represents probability of a specific interaction based on the CRAPome (**c**) Total unique spectra of GSK3α and GSK3β for tivantinib and ampicillin control pulldowns. (**d**) Western blot of GSK3α and GKS3β following drug affinity chromatography experiments with c-(−)-tivantinib and c-(+)-tivantinib in HL60 and U937 cells. Competition experiments were performed with 20 μM BIO. TCL = total cell lysate, BB = blocked beads

To confirm tivantinib’s ability to bind and inhibit GSK3α/β in these cells, we performed drug affinity chromatography using a couplable (−)-tivantinib analog as previously described (Fig. 2a)^13^. Pulldowns were of high quality with good reproducibility between biological replicates (Fig. S3a). To identify selective and potent interactions, we performed Significance Analysis of Interactomes (SAINT)^23,31^ analysis and determined the relative protein abundance in the sample eluates using the Normalized Spectral Abundance Factors (NSAF)^32^, respectively. In addition, we filtered the data set for common binders using the Contaminant Repository of Affinity Purification-Mass Spectrometry Data (CRAPome)^22^. We prioritized targets with a SAINTScore ≥ 0.95, a CRAPomePCT ≥ 0.95 and an –ln(NSAF) ≥ −7 (Fig. S3b). These criteria suggested two major kinase targets of tivantinib in these cells, namely GSK3α and GSK3β (Fig. 2b,c). Tivantinib selectivity was confirmed by Western blot, where (−)-c-tivantinib much more prominently enriched GSK3α/β as compared to (+)-c-tivantinib. Importantly, the GSK3 inhibitor BIO was able to compete away GSK3α/β suggesting a specific interaction (Fig. 2d). MET protein was not observed by proteomics in these cells.

### Tivantinib inhibits GSK3α/β signaling in AML cells

**Figure 3:**
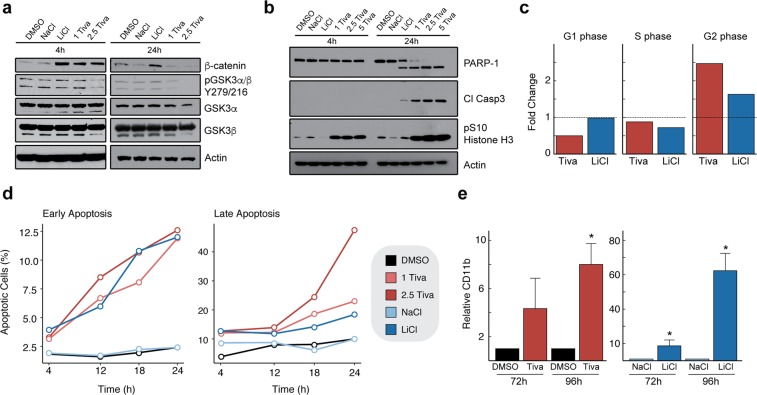
Analysis of cellular response following tivantinib treatment. (**a**) Effects of (-)-tivantinib (in μM), NaCl (20 mM) and the pan-GSK3 inhibitor LiCl (20 mM) on β-catenin and pGSK3α/β Y279/216 levels in HL60 cells. (**b**) Effects of tivantinib (in μM), NaCl (20 mM), and LiCl (20 mM) on PARP-1 and caspase 3 cleavage as well as pSer10 histone H3 levels after 4 and 24 h. (**c**) Cell cycle analysis by DAPI DNA staining following treatment of HL60 cells with DMSO, NaCl (20 mM), LiCl (20 mM), or tivantinib (1 μM) for 24 h. (**d**) Analysis of early and late apoptotic populations by Annexin V staining following treatment of HL60 cells for 4, 12, 18, or 24 h with DMSO, tivantinib (in μM), NaCl (20 mM), or LiCl (20 mM). (**e**) Cellular differentiation of HL60 cells following treatment with DMSO, tivantinib, NaCl or LiCl for 72 and 96 h as assessed by CD11b staining. Asterisk denotes p < 0.05 (*). Tiva = tivantinib.

To gain further insight into the downstream effects of GSK3 inhibition by tivantinib, we investigated the cellular outcome following drug treatment. Treatment of HL60 cells with tivantinib decreased GSK3α/β phosphorylation on Tyr279/216, which as an autophosphorylation site is directly correlated with GSK3 kinase activity (Fig. 3a)^33^. Furthermore, upon treatment with tivantinib we observed an increase in total β-catenin levels, which is characteristic for GSK3 inhibitors^6^. A larger and more prolonged increase in β-catenin was observed with LiCl than with tivantinib (Fig. 3a). Since β-catenin stabilization requires inhibition of both GSK3α and GSK3β^34^, this is consistent with LiCl strongly targeting both GSK3α/β isoforms and tivantinib being more selective for GSK3α, as we have shown previously by *in vitro* kinase assay^13^.

Previous studies have suggested that tivantinib causes G2/M arrest through inhibition of microtubule polymerization^30^, an observation which could also be explained by GSK3 inhibition. We therefore investigated the effects of tivantinib and LiCl on cell cycle arrest. Tivantinib caused a pronounced and rapid increase in phosphorylation of histone H3 Ser10 (Fig. 3b), which is indicative of cell cycle arrest. Detailed flow cytometry analysis showed a strong accumulation of cells in G2/M phase upon tivantinib treatment (Figs 3c, S4). This was similarly prominent with LiCl suggesting the observed G2/M arrest is mediated through inhibition of GSK3α/β. Furthermore, after 24 h of tivantinib treatment, we observed a strong and dose-dependent induction of apoptosis as assessed by PARP-1 and caspase 3 cleavage (Fig. 3b). Consistent with previous reports^5^, this was also apparent for LiCl although less pronounced than for tivantinib, even at relatively low doses. We next assessed the timing and magnitude of the induction of apoptosis by Annexin V staining followed by flow cytometry. Similar increases in early apoptosis were observed over time between tivantinib and LiCl; however, a much larger late apoptotic population was observed with tivantinib treatment (Figs 3d, S5).

Given that GSK3α silencing by RNAi has been described to induce cell differentiation^5^, we stained HL60 cells with α-CD11b to assess the ability of tivantinib and LiCl to differentiate AML cells by flow cytometry. Interestingly, while tivantinib treatment for 96 hours resulted in a significant increase of cell differentiation, LiCl caused a much stronger effect (Figs 3e, S6), which is consistent with previous studies^5^. Thus, tivantinib and LiCl have largely similar effects on AML cells as they both induce apoptosis, G2/M arrest, and differentiation. However one notable distinction is that tivantinib more potently induces apoptosis while LiCl has a markedly larger effect on cell differentiation.

### Tivantinib displays drug synergy with the BCL-2 inhibitor ABT-199

**Figure 4:**
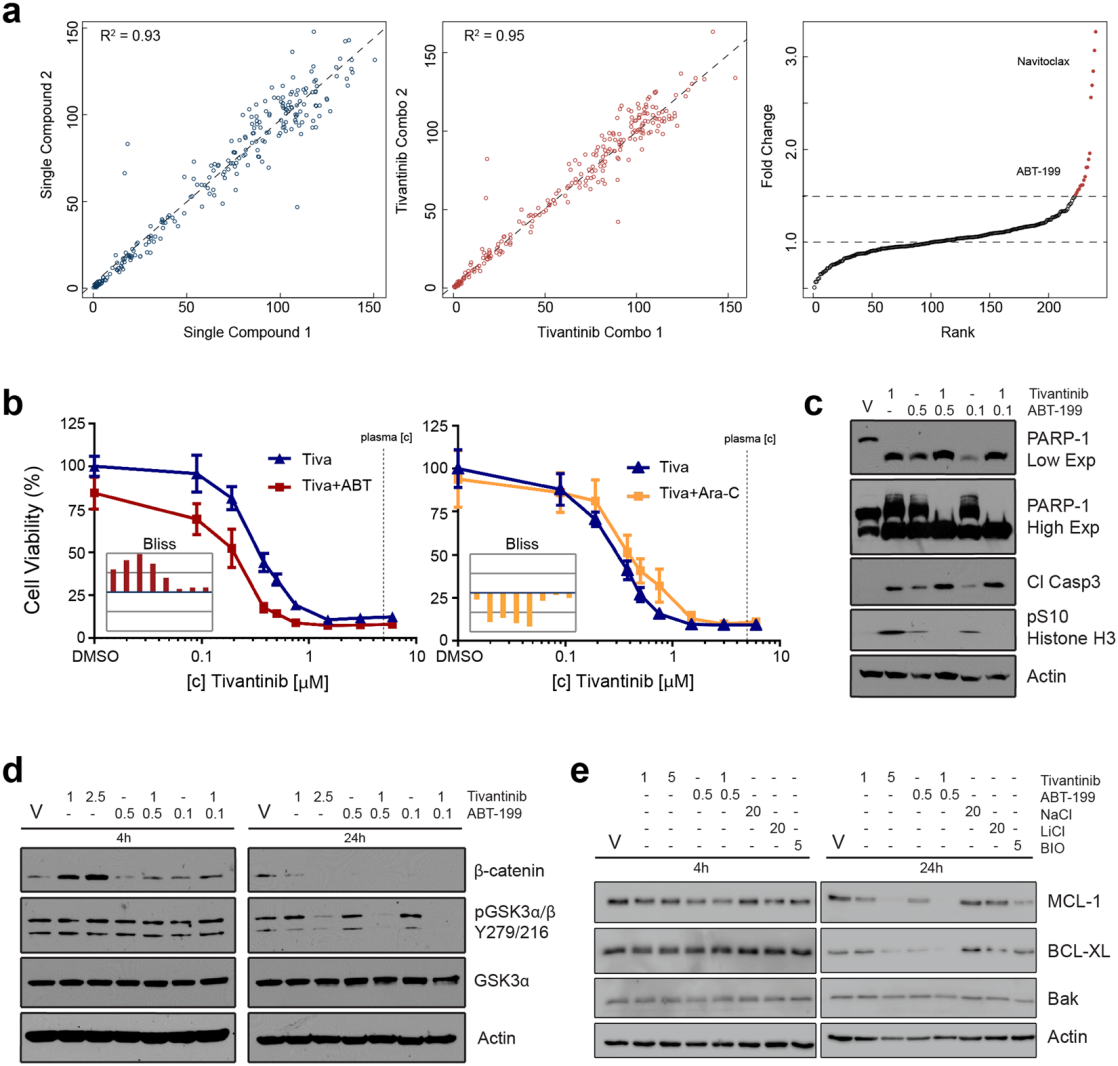
Identification of tivantinib and ABT-199 as a synergistic drug combination in AML cells. (**a**) Results of tivantinib combination drug screen in HL60 cells using a customized library of 240 targeted agents. Replicate correlations of cell viability following treatment with individual library compounds (2.5 μM) (left) and compounds in combination with tivantinib (0.25 μM) (middle) are displayed. Fold change corresponds to the ratio of inhibition of cell viability achieved by a drug combination with tivantinib (0.25 μM) compared to individual single library compounds (2.5 μM). Drugs passing fold change >1.5 cutoff are highlighted in red. Navitoclax and ABT-199 are labeled. (**b**) Dose response curves for inhibition of viability of HL60 cells of tivantinib and its combination with either ABT-199 (left) or cytarabine (Ara-C; right). Synergy is assessed by the Bliss model of independence (histograms in insets). Displayed in the histograms are the experimentally determined differences for each drug combination from the calculated Bliss additivity on a scale of +20% to −20% cell viability in order of increasing tivantinib concentrations. Vertical lines indicate increments of 10% cell viability. Bars pointing up from the blue baseline (additivity) indicate synergy, bars pointing down indicate antagonism. (**c**) Effects of tivantinib and ABT-199 combination (in μM) on PARP-1 and caspase 3 cleavage as well as pSer10 histone H3 levels after 24 h treatment. (**d**) Effects of tivantinib and ABT-199 combination on β-catenin stabilization and pGSK3α/β Y279/216 levels. (**e**) Effects of tivantinib and ABT-199 combination on MCL-1, BCL-XL, and Bak. V = vehicle (DMSO). Tivantinib, ABT-199, and BIO concentrations are in μM. NaCl and LiCl concentrations are in mM.

Resistance against single drug therapy with targeted agents can often be delayed or suppressed by potent drug combinations. In the case of tivantinib, we hypothesized that drug combinations may allow for a reduction of the tivantinib dose and thereby a less pronounced stabilization of β-catenin. In order to further amplify tivantinib’s anticancer activity in AML cells, we conducted a drug combination screen in HL60 cells using a collection of 240 clinically relevant targeted agents. The majority of these (90+%) were in clinical development so that identification of a drug that synergizes with tivantinib would have the potential for clinical translation. The data was highly reproducible with good correlations between biological replicates (Fig. 4a). One of the strongest hits from this screen for potential synergy with tivantinib in HL60 cells was the BCL-2 inhibitor navitoclax (ABT-263) (Fig. 4a). This was interesting as cancer cell lines with activating mutations in β-catenin or increased β-catenin levels as the result of GSK3 inhibition have been shown to exhibit increased sensitivity to BCL-2 inhibitors^35^. In addition to navitoclax, we identified its newer structural analogue ABT-199 (venetoclax) as a potentially synergistic drug (Fig. 4a)^36^. We posited that since navitoclax has shown acute toxicity in patients and ABT-199 has been recently approved by the FDA for chronic lymphocytic leukemia, combination of tivantinib with ABT-199 may be a safer alternative with higher translational potential^37^. Importantly, ABT-199 has already been shown to be effective in AML cells^38,39^, leading to a recent FDA designation as a breakthrough therapy and multiple AML specific clinical trials currently recruiting patients to test the safety and efficacy of ABT-199 alone or in combination with chemotherapy. We therefore selected ABT-199 for detailed synergy analysis. In addition to a clear shift of the dose response curve for the combination treatment, synergy analysis using the Bliss model of independence across a range of concentrations suggested pronounced synergy within physiologically relevant concentrations between tivantinib and ABT-199, but antagonism with the standard of care agent cytarabine (Ara-C) (Fig. 4b).

As we had observed a strong induction of apoptosis with tivantinib treatment and as ABT-199 inhibits the anti-apoptotic protein BCL-2, we hypothesized that combining tivantinib and ABT-199 would also further increase apoptotic signaling. Indeed, upon combination treatment of tivantinib and ABT-199, we observed an increase in cleavage of caspase 3 and a complete cleavage of PARP-1 protein, in which no native 116 kDa PARP-1 remained, suggesting a large increase in apoptosis (Fig. 4c). The pronounced G2/M arrest as indicated by pS10 histone H3 following tivantinib treatment was reversed by the drug combination. ABT-199 by itself did not affect GSK3 tyrosine phosphorylation, but the combination with tivantinib caused complete loss of pY GSK3 (Fig. 4d). Importantly, addition of ABT-199 completely abrogated the increase of β-catenin that is observed with single agent tivantinib treatment (Fig. 4d). Since MCL-1 and BCL-XL expression have been shown to cause resistance to ABT-199^39,40^, we next hypothesized that the observed synergy with tivantinib was a result of altered MCL-1 and BCL-XL protein levels. Interestingly tivantinib single agent and, more pronouncedly, ABT-199 combination caused a loss of anti-apoptotic MCL-1 and BCL-XL protein levels while maintaining pro-apoptotic Bak levels (Fig. 4e). In summary, these results suggest that tivantinib and ABT-199 combination greatly increases the already strong apoptotic effects of tivantinib in AML cells by down regulating anti-apoptotic proteins while simultaneously suppressing activation of β-catenin.

### The combination of tivantinib and ABT-199 is effective in primary AML patient samples

**Figure 5:**
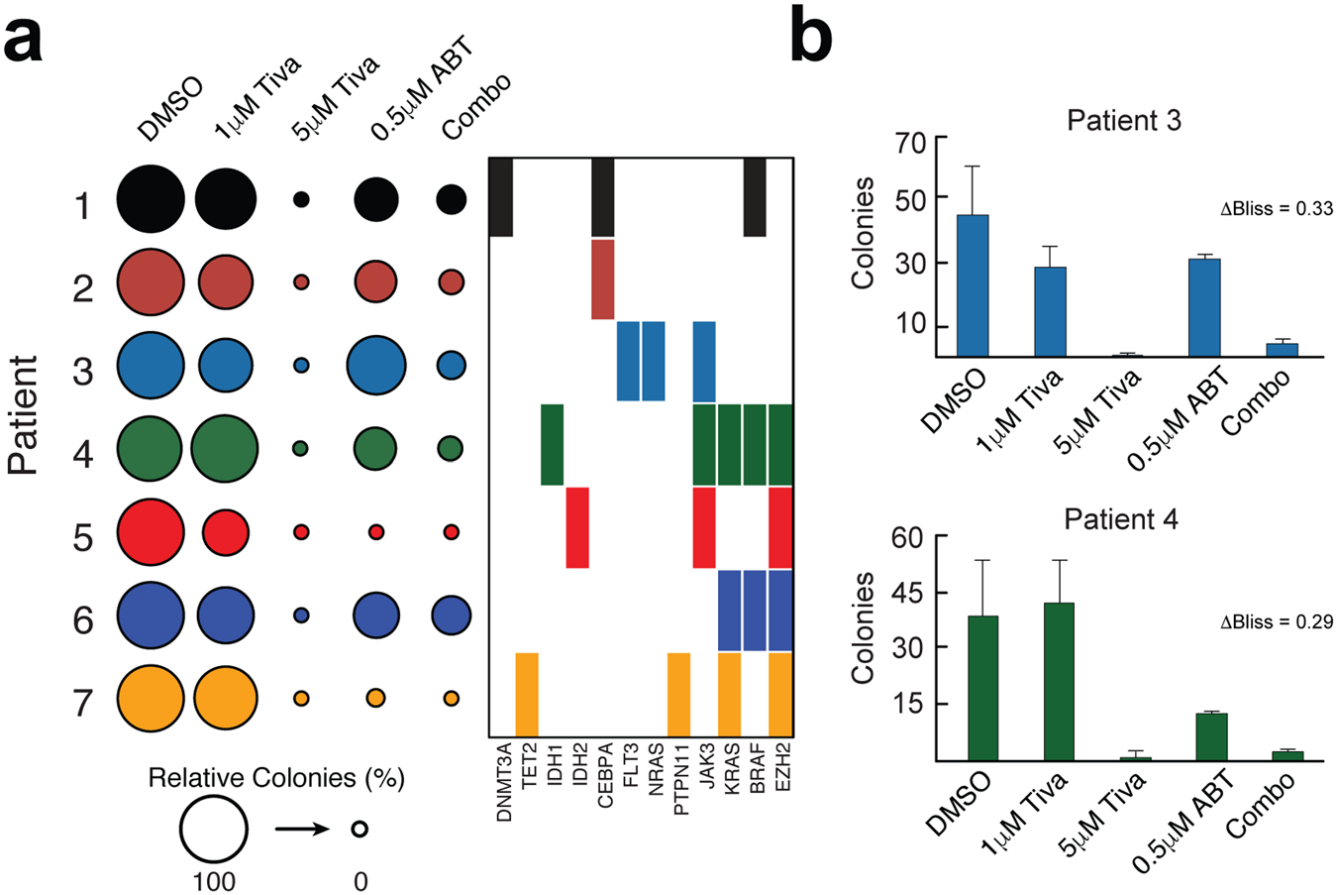
Effect of tivantinib, ABT-199 and their combination on primary AML patient blasts. (**a**) Dotplot of relative primary AML BMNCs colony formation following treatment with tivantinib, ABT-199 or their combination for 14 or 19 days. Counts were averaged and normalized to DMSO. Patient mutational status for commonly altered genes is displayed. (**b**) Absolute primary AML blast colony count for patients 3 and 4 following treatment for 19 and 14 days, respectively. Synergy values (deviation from Bliss) are annotated. Combo = 1 μM tivantinib + 0.5 μM ABT-199.

In order to better evaluate the potential for clinical translation of our observations with tivantinib in AML, we next tested the efficacy of tivantinib in primary AML patient Bone Marrow Mononuclear Cells (BMNCs) as a single agent, as well as in combination with ABT-199. Using several different primary AML patient samples, tivantinib displayed a strong ability to inhibit colony formation at the clinically relevant concentration of 5 μM across all patients with only a few colonies remaining (Figs 5a, S7). Single drug ABT-199 treatment showed slight variations in efficacy, but on average reduced colony formation to approximately 30–40 percent consistent with previous reports (Figs 5a, S7)^39,40^. The combination of tivantinib and ABT-199 exerted synergy in 4 of 7 patients with moderate (patients 1 and 2) to strong synergy (patients 3 and 4) values (Figure S7). Patients 5 and 7 were exquisitely sensitive to ABT-199 single agent treatment and therefore a Bliss value could not be accurately calculated. Consistent with GSK3α being overexpressed across all major AML karyotypes (Figure S1a), tivantinib efficacy did not show any obvious relationship with mutational status or karyotypes (Fig. 5a; Table S2) although the number of patient samples was low. Overall, these data suggest that tivantinib is highly effective in inhibiting the colony forming capacity of primary AML patient samples as a single agent or in combination with ABT-199.

## Discussion

GSK3 plays a central role in a broad range of cellular processes, including glycogen metabolism, insulin signaling, apoptosis and microtubule function. Accordingly, it is under investigation as a potential target in Alzheimer’s disease and diabetes^6,41^. In the context of cancer, GSK3 is best known in its function as a tumor suppressor, which is deactivated by AKT or Wnt signaling^6,41^. However, most studies have focused on GSK3β, whereas significantly less is known about GSK3α. Moreover, it is increasingly appreciated that GSK3 signaling is context-dependent. For instance, there have been several reports that describe tumor supporting roles of GSK3α in glioblastoma, pancreatic cancer, multiple myeloma, and MLL-rearranged leukemia^28,42–44^.

GSK3α has been identified by Banerji *et al*. through a functional genomic screen as a promising target in AML^5^. However, targeting GSK3 in hematological malignancies does have theoretical challenges in that GSK3 is known to phosphorylate β-catenin thereby marking it for subsequent proteasomal degradation. Upon GSK3 inhibition, β-catenin accumulates, translocates to the nucleus and activates transcriptional pathways^6,45^. Such increased β-catenin signaling has been implicated in a number of leukemogenic effects, such as self-renewal of leukemic stem cells^46^. As β-catenin stabilization requires inhibition of both kinases and most GSK3 inhibitors target GSK3α and GSK3β with similar potency^34^, these compounds may possess some significant limitations. Accordingly, the nonspecific pan-GSK3 inhibitor LiCl, which is currently FDA approved for the treatment of epilepsy and bipolar disorder^7,8^, has met limited success in clinical studies of AML^47–49^. In addition to isoform selectivity, various other aspects, such as the binding mode, magnitude of inhibition, and kinome-wide target specificity influence the overall cellular outcome of inhibiting GSK3α. For this reason, not all GSK3 inhibitors should be treated equally.

Tivantinib was originally developed as an inhibitor of the receptor tyrosine kinase MET^14^, but its target selectivity and mechanism of action has been a controversial subject. We and other groups have suggested that MET is actually not a significant target of tivantinib in many cancer cells^13,30,50^. We have previously observed that tivantinib, although being indeed a weak MET inhibitor, much more prominently targets GSK3α and GSK3β in NSCLC cells and that inhibition of these targets can explain its potent anticancer activity in NSCLC^13^. Importantly, we also noted remarkable kinome-wide specificity and some moderate selectivity of tivantinib for GSK3α over GSK3β, which are unique features among clinical GSK3 inhibitors^12^. It has also been suggested that tivantinib binds tubulin and inhibits microtubule dynamics resulting in anticancer activity^30,50^. While these observations are compelling, our results suggest that the sensitivity profile of tivantinib in AML cells more closely matches that of a GSK3 inhibitor. Furthermore, we show that pharmacological inhibition of GSK3 by LiCl largely mimics tivantinib’s effects in these cells with regard to viability, apoptosis, cell cycle arrest and differentiation. However, as GSK3α/β have well described roles in microtubule regulation through phosphorylation of Tau (MAPT)^51,52^ and other microtubule associated proteins, such as MAP2C^53^, it would be difficult to precisely identify the contributions to tivantinib’s overall cellular effects that stem from targeting tubulin in addition to GSK3, particularly as histone H3 phosphorylation is induced rapidly, which could indicate further crosstalk between GSK3 and the cell cycle regulation pathway^54^. Impairment of microtubule polymerization however may be translationally beneficial as it may result in synergistic anticancer activity in the context of dual GSK3 and BCL-2 inhibition as has been previously observed in breast cancer^55^. However, additional studies are necessary to elucidate the complex interplay between these pathways in AML.

In light of the suggested role of GSK3α in AML, we investigated the potential for repurposing tivantinib for the treatment of AML, which to the best of our knowledge has not been reported. Consistent with previous reports^5^, analysis of publically available datasets showed that GSK3α is overexpressed in AML and that knockdown of GSK3α has strong effects on the viability of AML cell lines. We also show that tivantinib interacts with and inhibits GSK3α/β in AML cells and that it potently kills these cells by inducing apoptosis. Interaction of tivantinib with its intended target MET, which was observed to a minor extent in NSCLC cells^13^, was not detectable in AML cells. While tivantinib does target both GSK3α and GSK3β, its effects on β-catenin levels were somewhat less pronounced than with LiCl. This was consistent with our previous observation that tivantinib is more selectively targeting GSK3α^13^. Also, β-catenin stabilization was more transient with tivantinib, whereas it is sustained for a longer period of time upon LiCl treatment. Tivantinib may therefore provide an important therapeutic advantage over pan-GSK3 inhibitors, such as LiCl. Banerji *et al*. have shown that LiCl readily causes differentiation of AML blasts at relatively low concentrations^5^. Our results confirmed these observations and showed that tivantinib also induces differentiation. However, tivantinib was a much stronger inducer of apoptosis than of differentiation; and although LiCl also induces apoptosis, tivantinib is markedly more potent than LiCl in this regard, which might be due to additional effects of tivantinib on microtubules.

In addition to tivantinib exhibiting potent single agent activity in AML, we observed that the BCL-2 inhibitor ABT-199, which displays activity and is in clinical trials in AML^39^, synergizes with tivantinib by further enhancing tivantinib’s already potent ability to inhibit cell viability and induce apoptosis. MCL-1 and BCL-XL expression have previously been associated with ABT-199 resistance^39,40^, and it is noteworthy that we observed a dose-dependent decrease in MCL-1 and BCL-XL levels following tivantinib treatment, which was enhanced in combination with ABT-199. This is consistent with previous reports showing that tivantinib downregulates these proteins in hepatocellular carcinoma^56^. It is also known that GSK3 transcriptionally regulates BCL-XL expression and that GSK3 inhibition by BIO or SB-415286 leads to a reduction in BCL-XL levels^57,58^. Interestingly, while BIO similarly downregulated BCL-XL in breast cancer cells, it also reduced MCL-1 expression, not via transcriptional control, but through a proteasome-dependent mechanism^57^. This downregulation of MCL-1 and BCL-XL expression likely contributes to the synergy observed between tivantinib and ABT-199. By downregulating anti-apoptotic MCL-1 and BCL-XL that cause ABT-199 resistance, tivantinib is amplifying the relative apoptotic effect of ABT-199. This synergy is in excellent agreement with a previous study that described cancer cells with increased β-catenin levels, for instance as the consequence of GSK3 inhibition, to be particularly sensitive to inhibition of BCL-2 by the ABT-199 analogue navitoclax^35^. Interestingly, in addition to modulation of anti-apoptotic proteins we observed that the tivantinib/ABT-199 combination completely abrogated β-catenin stabilization seen with tivantinib single drug treatment. This was apparent already after 4 hours and is therefore likely due to cross-talk between the GSK3 and BCL-2 pathways that is independent of altered transcription. This pronounced reduction of β-catenin persisted for 24 hours and could possibly help prevent some of the leukemogenic effects previously associated with β-catenin signaling in AML^46^. Thus by modulating tivantinib’s effects on β-catenin levels in conjunction with the amplification of apoptotic signaling, this suggests a superior therapeutic potential of this drug combination in AML.

In this context, it is important to note that tivantinib, as a single drug and even stronger in combination with ABT-199, showed potent anti-leukemic activity in AML patient-derived samples within clinically relevant concentrations. This appears to be independent of the mutational status of common prognostic genes although our sample size was too small to allow broader conclusions. A potential correlation with tivantinib sensitivity could be amplification of GSK3α expression levels as GSK3α is more highly expressed in several different subtypes of AML, including *11q23* MLL-rearranged leukemia, which has previously been shown to be sensitive to GSK3 inhibition^13,28^. Interestingly, high expression of GSK3 and BCL-XL has previously been suggested to correlate with poor prognosis in AML^59^. However, a more thorough investigation of GSK3α expression and signaling in AML is necessary to make detailed conclusions. Although tivantinib has been described to cause myelosuppression^60,61^, this is readily managed in most cases and tivantinib is generally considered a well-tolerated compound^60,61^, which has been evaluated in more than 40 clinical studies, including phase III. Considering also that the concentrations required for its activity in AML cells are well within the therapeutically achievable levels and could be potentially even further reduced in combination with ABT-199^61,62^, repurposing tivantinib provides a tangible opportunity for clinical translation into AML.

In summary, repurposing the advanced clinical drug candidate tivantinib based on its off-target GSK3α identified it as a highly potent agent in AML cells. Combination with the BCL-2 inhibitor ABT-199, which is already under clinical investigation for AML, further enhanced tivantinib’s potency and eliminated undesirable β-catenin activation. Together, these findings suggest that tivantinib, either as a single agent or in combination with ABT-199, represents a novel and promising therapeutic option for AML, a disease, which is still in high need for new therapies.

## Supplementary information

Supplementary Information

Supplementary Table S1

Supplementary Table S2

## Data Availability

The mass spectrometry proteomics data have been deposited to the ProteomeXchange Consortium via the PRIDE partner repository with the dataset identifiers PXD010217 and 10.6019/PXD010217^63^.
