# Supplementary material for: Off-target based drug repurposing opportunities for tivantinib in acute myeloid leukemia
Source: Sci Rep. 2019 Jan 24;9:606. doi: 10.1038/s41598-018-37174-6 (PMC6345777; doi:10.1038/s41598-018-37174-6)

Supplementary Information

Off-target based drug repurposing opportunities for tivantinib in acute myeloid leukemia

Brent M. Kuenzi,[1,5,#] Lily L. Remsing Rix,[1,#] Fumi Kinose,[3] Jodi L. Kroeger,[4] Jeffrey E. Lancet,[2] Eric Padron[2] and Uwe Rix[1]*

[#]These authors contributed equally.

[1]Department of Drug Discovery, [2]Department of Hematologic Malignancies, [3]Department of Thoracic Oncology and [4]Flow Cytometry Core, H. Lee Moffitt Cancer Center & Research Institute, Tampa, Florida 33612, United States; [5]Cancer Biology Ph.D. Program, University of South Florida, Tampa, Florida 33620, United States

*Correspondence: Uwe.Rix@moffitt.org

Supplementary Information

Figure S1. GSK3α is an actionable target in AML. (**a**) Hierarchical coexpression tree of GSK3α gene expression across hematopoietic cell types. (**b**) Hierarchical coexpression tree of GSK3β gene expression across hematopoietic cell types. Size and color is associated with gene expression. (**c**) RSA sensitivity score of GSK3α/β shRNA sensitivity across AML cell lines. Wilcoxon-rank sum test was used to establish significance. (**d**) RSA sensitivity score of GSK3α shRNA sensitivity across hematopoietic cell lines. AML cell lines are highlighted in red.

Figure S2. Tivantinib sensitivity modeling and validation. (a) Receiver operator characteristic (ROC) curve of tivantinib sensitivity predictions across CTRP. (**b**) Lolliplot of feature importance for top 15 features selected during elastic net regularization. Displayed is the absolute value of the relative feature weights. Inset is a STRING network (medium confidence) of known associations of these gene features with GSK3 signaling. Non-feature genes included in the network are colored in black. (**c**) Bar plot comparing spearman correlation of AML sensitivity predictions for (-)-tivantinib with paclitaxel, SGX253 and ML320 from elastic net regularized regression. Highest possible spearman correlation based on tivantinib actual vs. predicted correlation (limit of reproducibility) is displayed.

Figure S3. Chemical proteomic characterization of tivantinib. (**a**) Correlation of total unique spectra between biological replicates of c-(-)-tivantinib pulldowns in HL60 cells. (**b**) Log2 Fold Change vs. SaintScore of drug affinity chromatography in HL60 cells. Red points indicate proteins passing 95% CRAPomePCT and abundance (NSAF) filtering. Red box indicates points passing SaintScore cutoff before kinase selection. GSK3α and GSK3β are labeled.

Figure S4. Cell cycle analysis following treatment of HL60 cells with tivantinib and LiCl. Representative histograms of DAPI DNA staining following treatment of HL60 cells with DMSO, NaCl (20 mM), LiCl (20 mM) or (-)-tivantinib (1 μM) for 4 or 24h.

Figure S5. Apoptosis levels following treatment of HL60 cells with tivantinib and LiCl. HL60 cells were treated with DMSO, tivantinib (1 or 2.5μM), NaCl (20mM) or LiCl (20mM) for 4, 12, 18 or 24h and apoptosis was assessed by Annexin V / DAPI staining of viable cells.

Figure S6. Cellular differentiation following treatment of HL60 cells with tivantinib and LiCl. HL60 cells were treated with DMSO, tivantinib, NaCl or LiCl at the indicated concentrations for 72 and 96h and differentiation was assessed by CD11b staining of viable cells.

Figure S7. Effect of tivantinib, ABT-199 and combination on primary AML patient blasts. Absolute primary AML blast colony counts for patients 1-7 following treatment for 19 or 14 days as indicated. Synergy values (deviation from Bliss) are annotated. ## represents a biological singlicate. Tiva = tivantinib; ABT = ABT-199. All concentrations are in μM.

Figure S8. Full western blot scans. Full western blot scans for figures 2, 3, and 4.

Supplementary Table 1. APOSTL analysis of tivantinib target profile. Raw APOSTL output of tivantinib drug affinity chromatography enrichments. Listed are the SAINTscore, log2(FoldChange), CRAPome percentage (probability of being a specific interaction based on CRAPome database) and ln(NSAF) parameters. Prey: identified protein (UniProtKB accession code); PreyGene: official gene name of identified protein; Spec: number of Exclusive Unique Spectral Counts (EUSC) per each biological replicate;

ctrlCounts: EUSC of control experiments per replicate. Ampicillin was used as a negative control. TIVA = c-(-)-tivantinib affinity enrichment.

Supplementary Table 2. Clinical characteristics of patients at time of diagnosis. WHO: World Health Organization classification; FAB: French-American British classification (M0-M7); WBC: white blood count (cellsx10^3/dL); HgB at Dx: Hemoglobin at Diagnosis (g/dL); Plt: platelet (cellsx10^3/dL); BMBx: bone marrow biopsy.

Kuenzi et al. Supplementary Figure 1

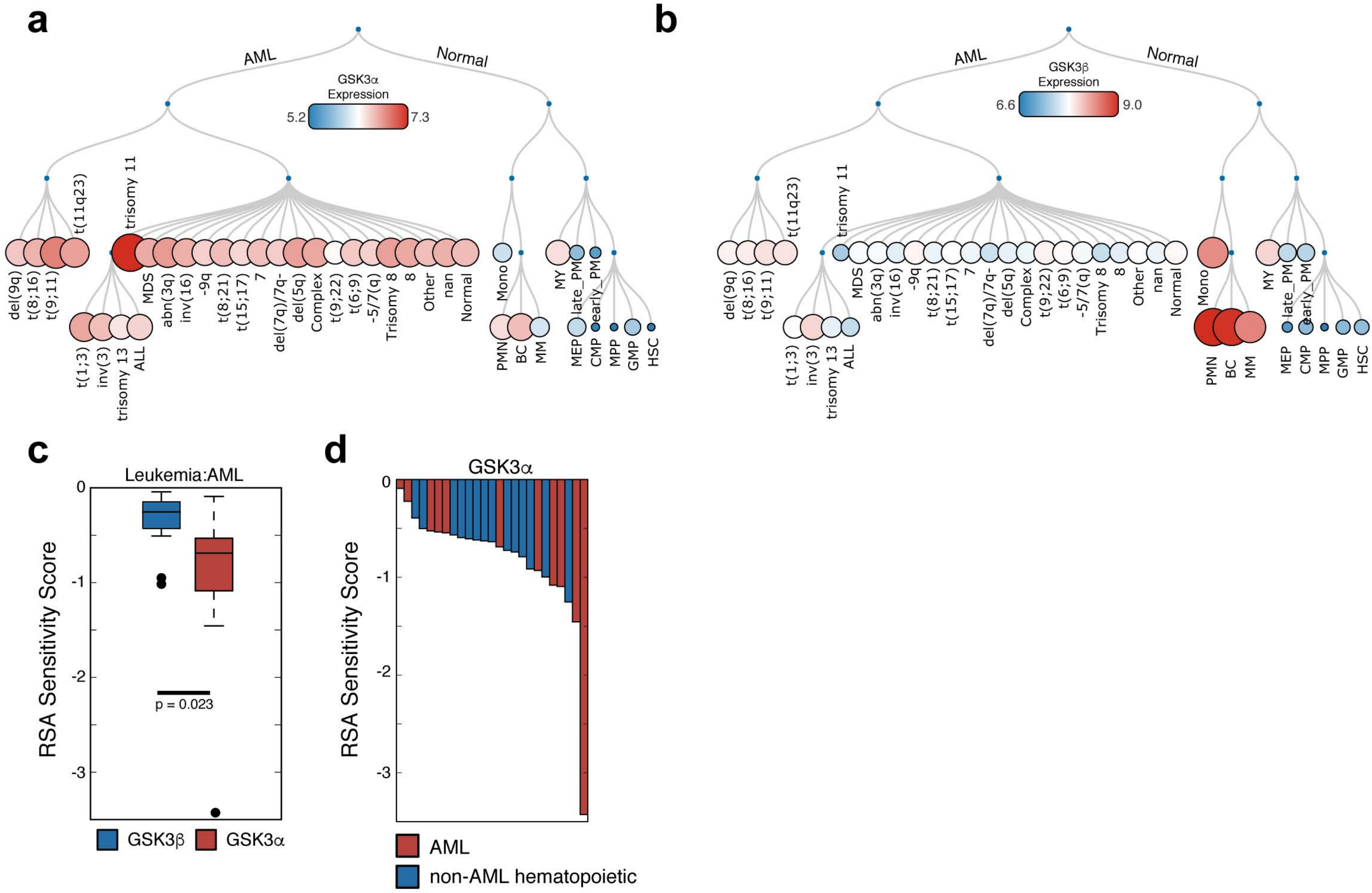

Kuenzi et al. Supplementary Figure 2

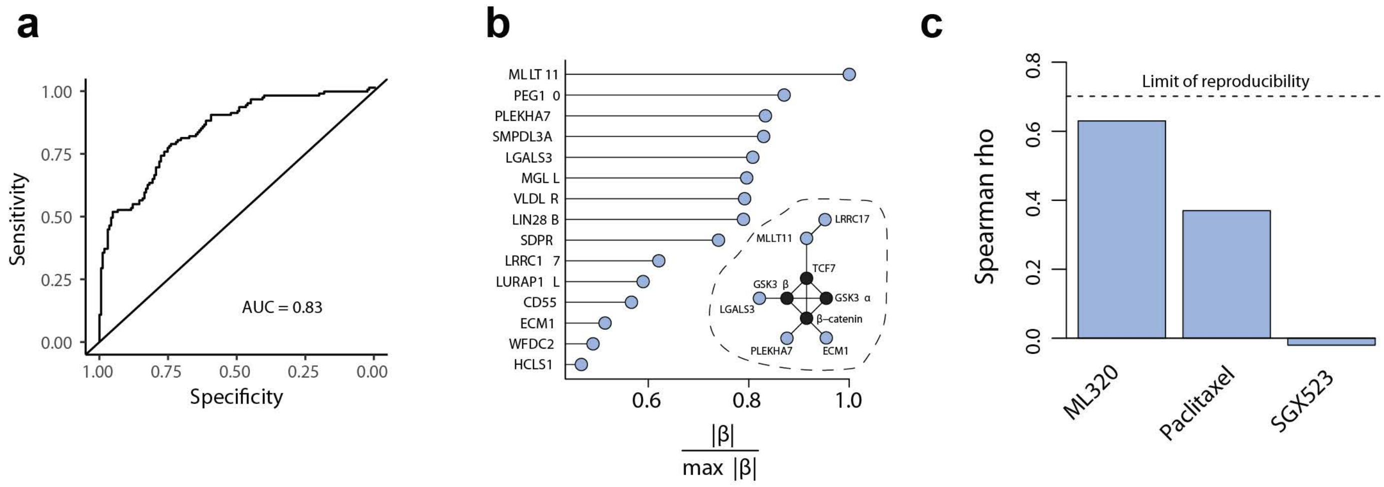

Kuenzi et al.   Supplementary Figure 3

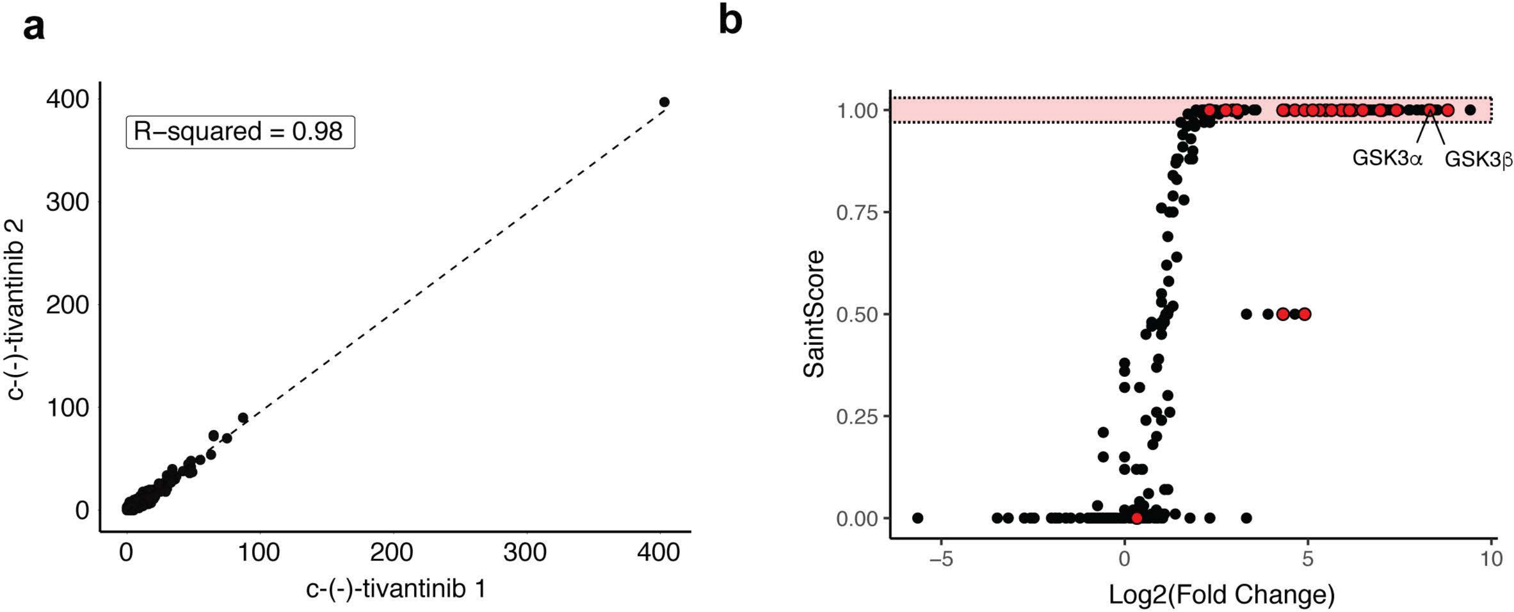

Kuenzi et al. Supplementary Figure 4

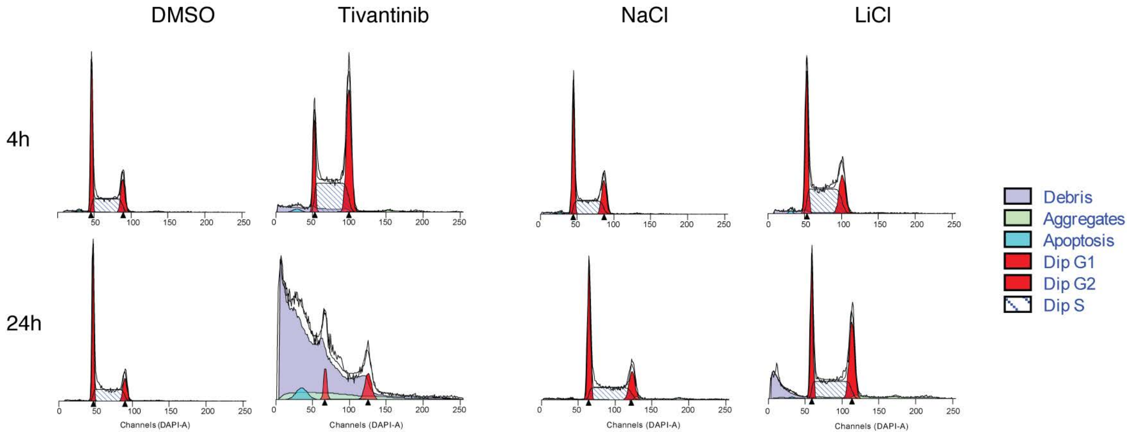

Kuenzi et al. Supplementary Figure 5

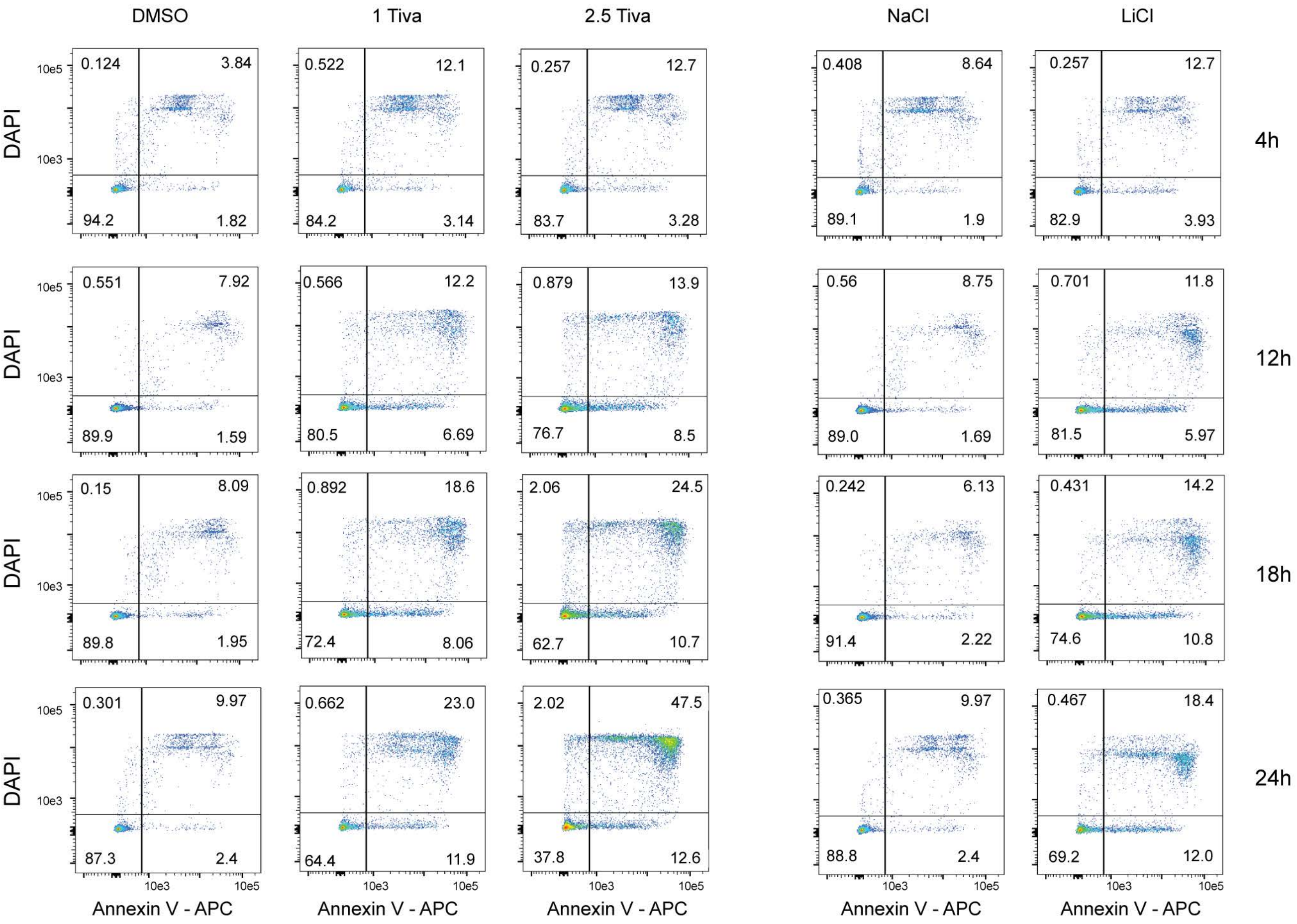

Kuenzi et al. Supplementary Figure 6

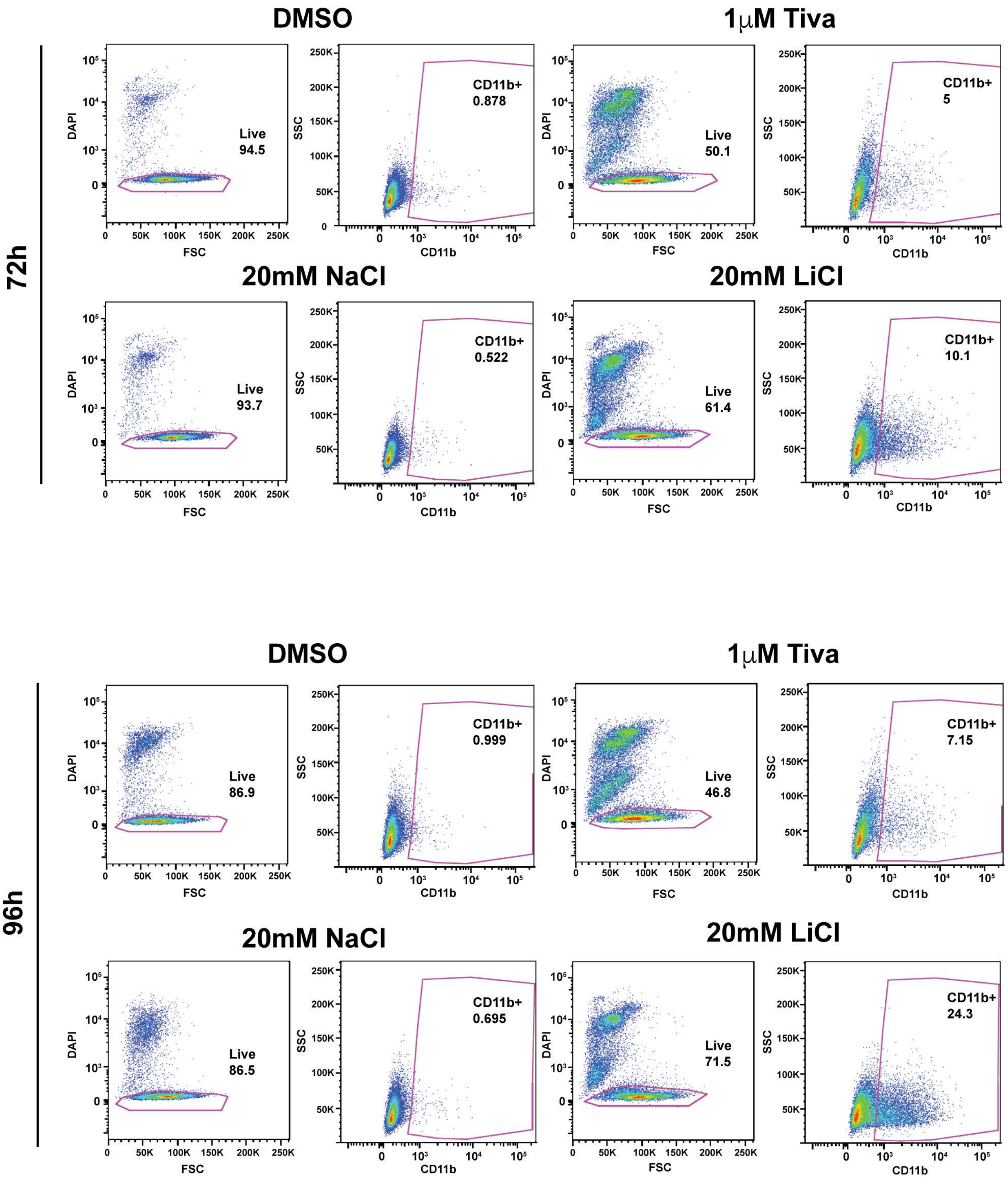

Kuenzi et al.   Supplementary Figure 7

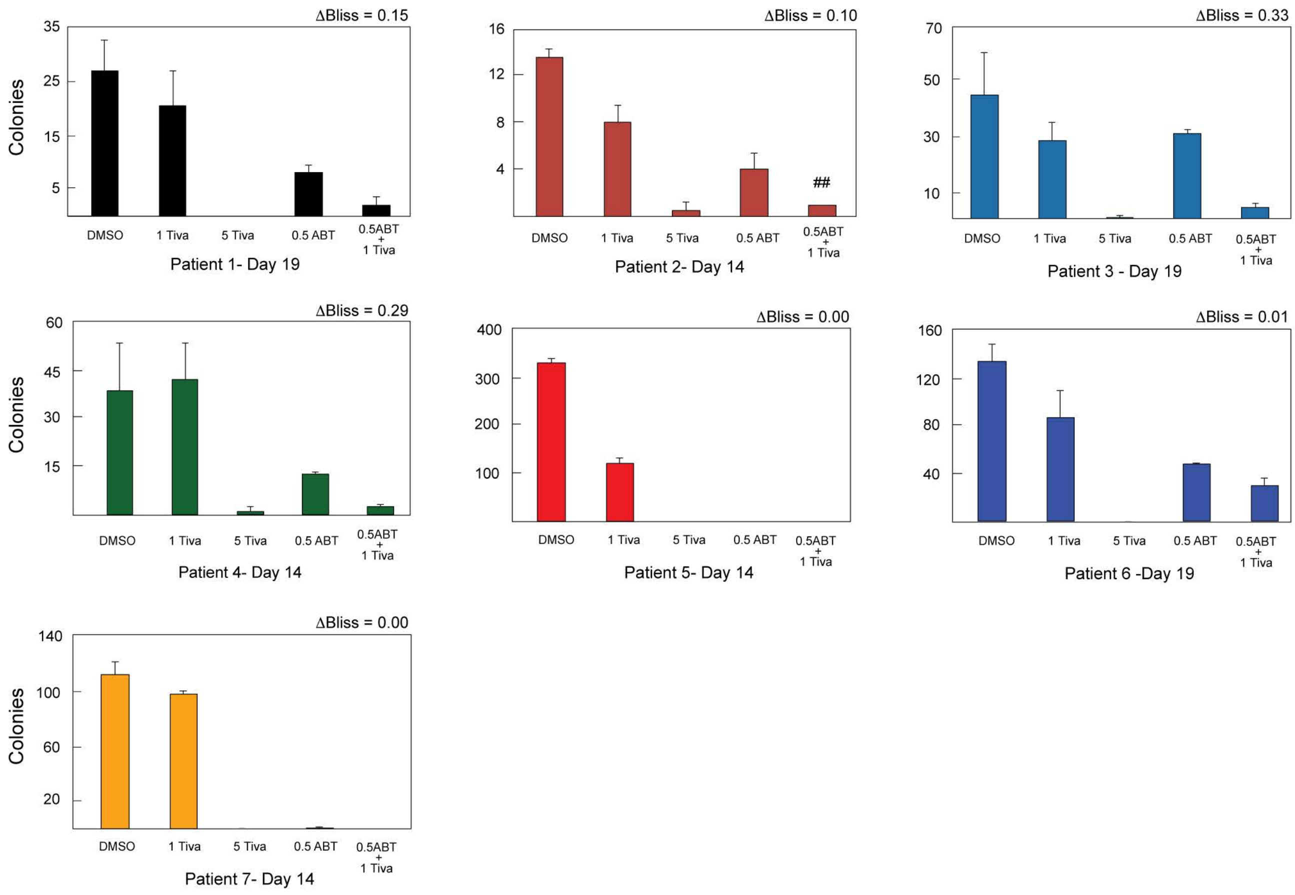

Kuenzi et al.   Supplementary Figure 8

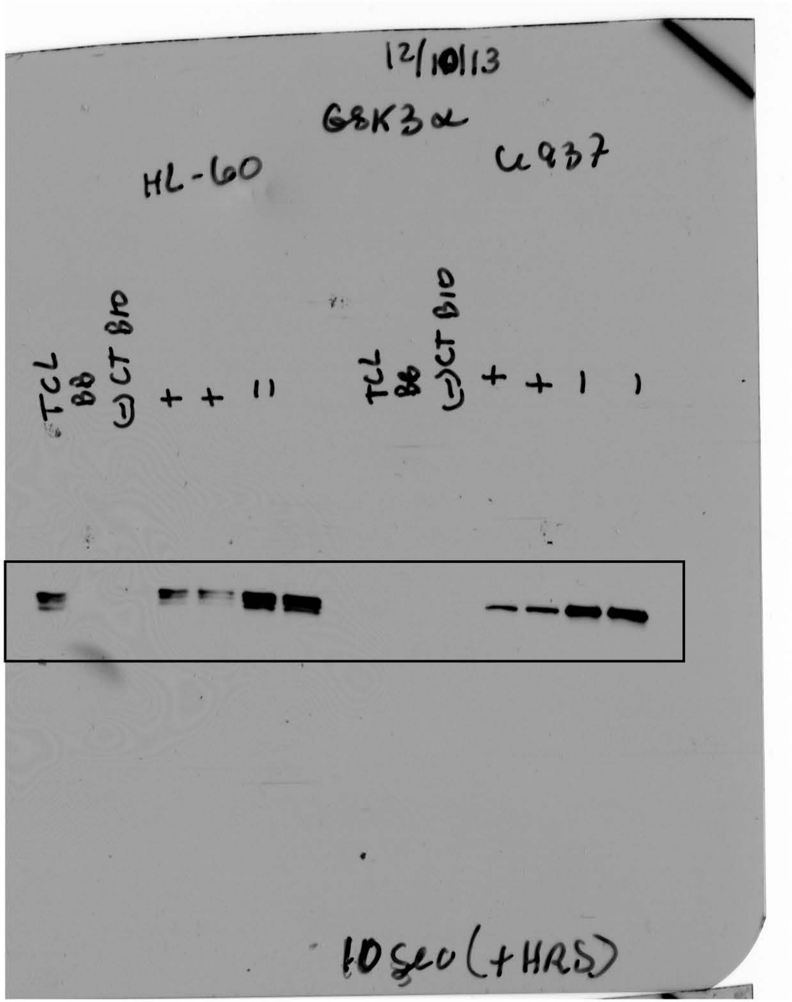

Figure 2d - GSK3a

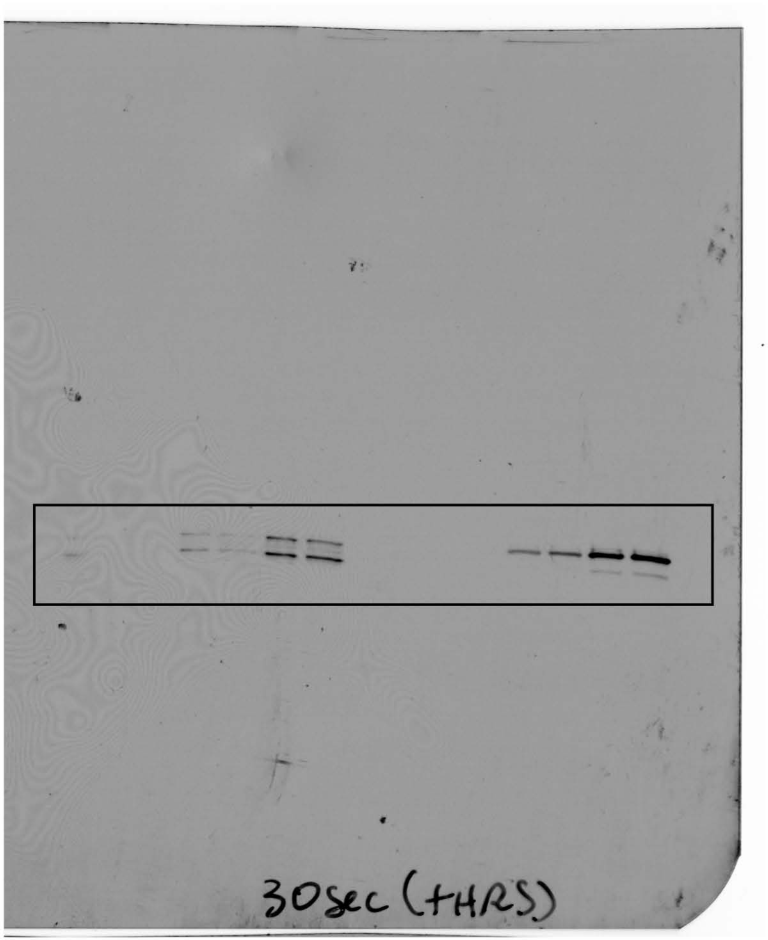

Figure 2d - GSK3b

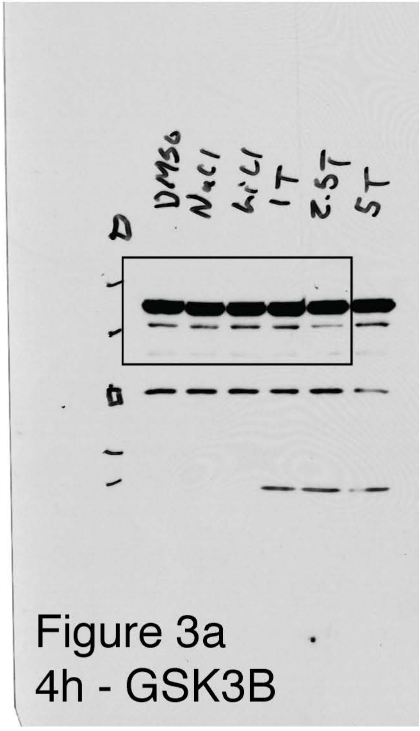

Figure 3a
4h - GSK3B

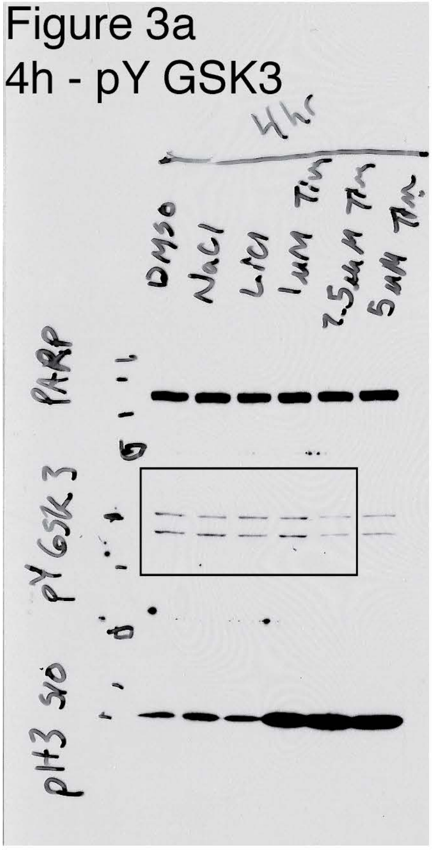

Figure 3a
4h - pY GSK3

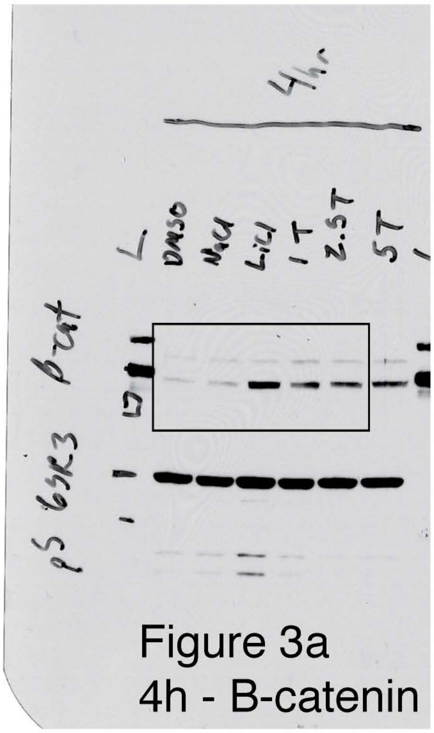

Figure 3a
4h - B-catenin

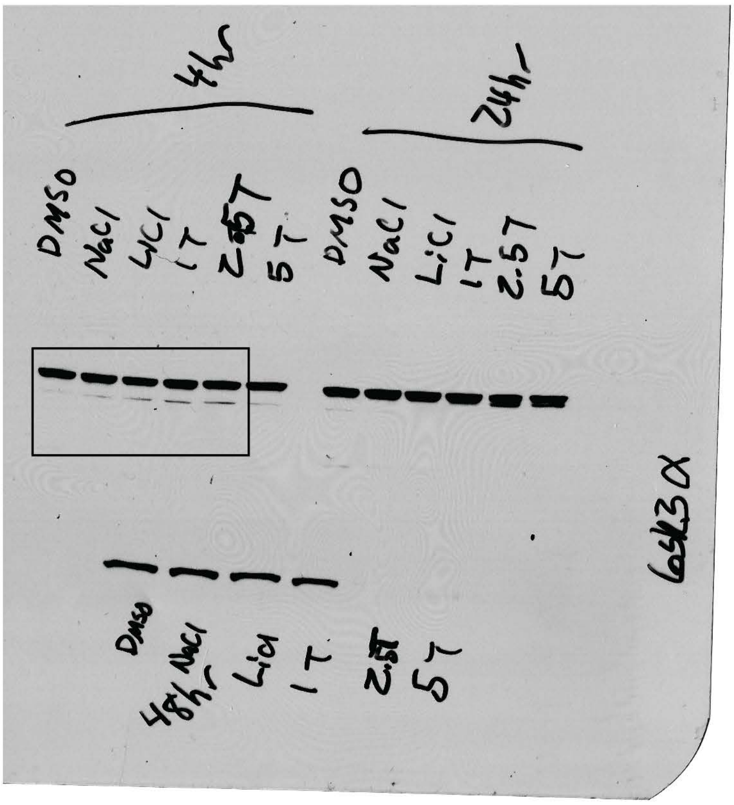

Figure 3a
4h - GSK3a

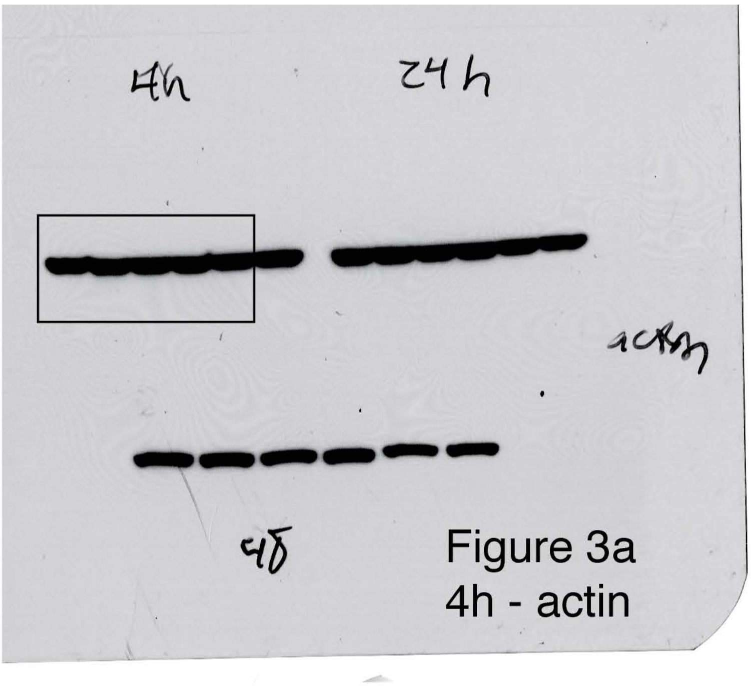

Figure 3a
4h - actin

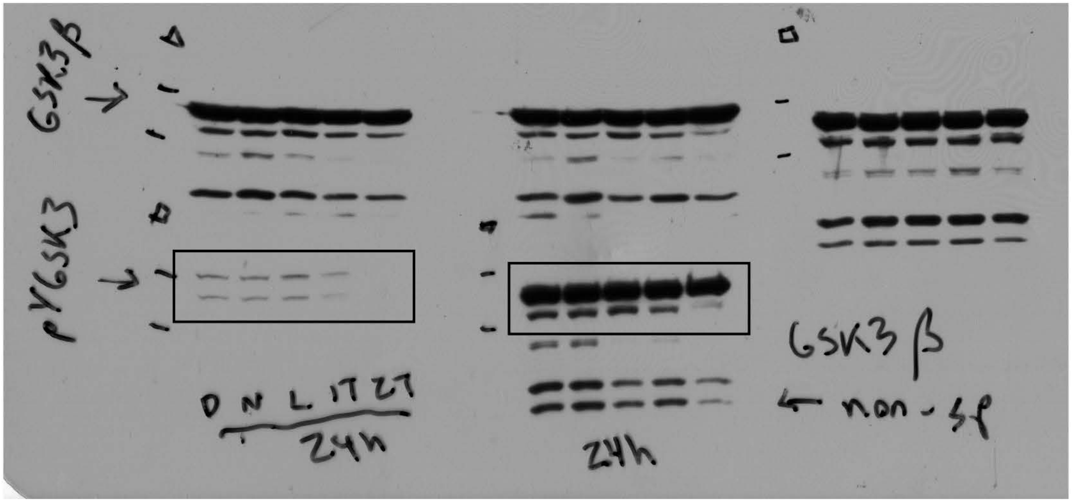

24h - pY GSK3

24h - GSK3B

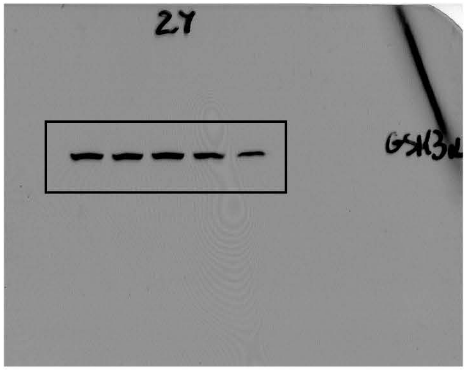

24h - GSK3a

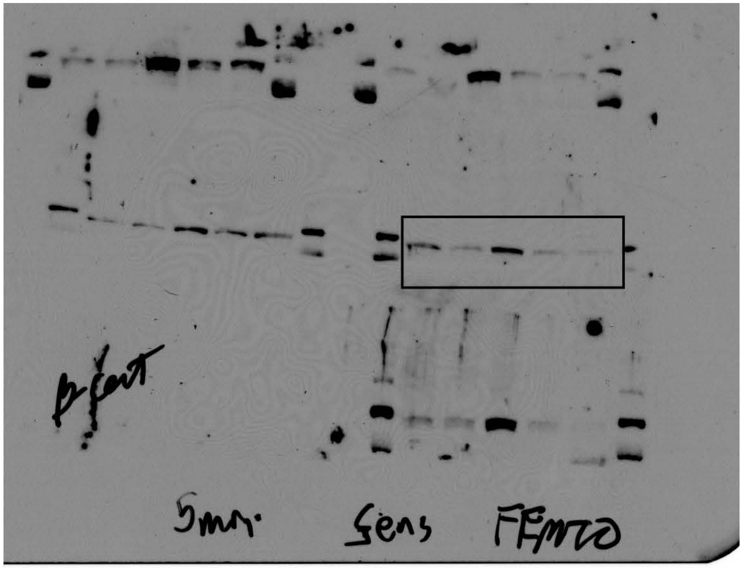

24h - B-catenin

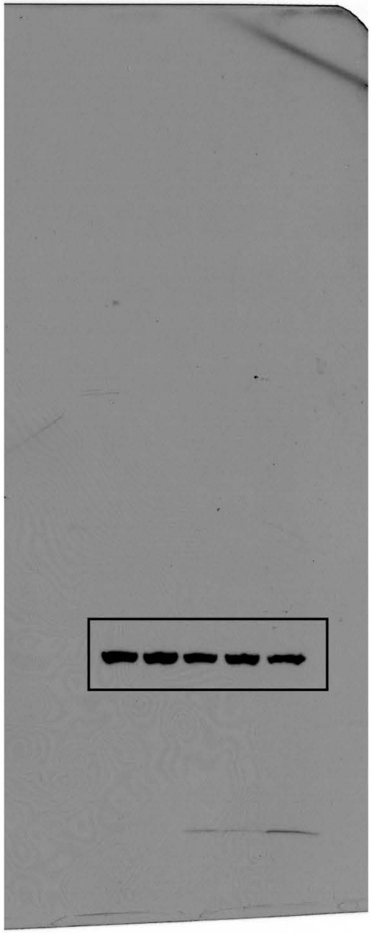

24h - actin

Figure 3a

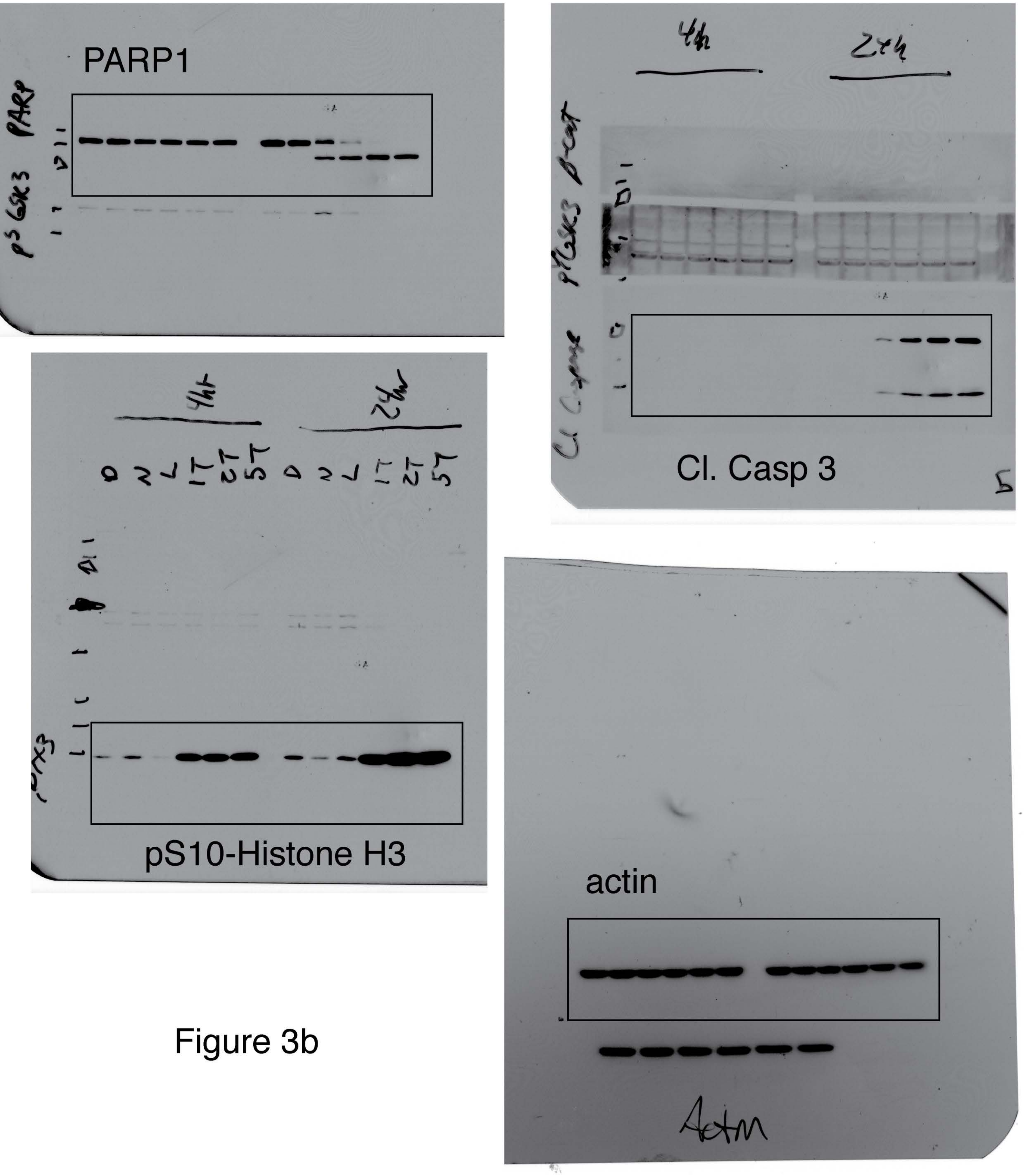

Figure 3b

Figure 4c - PARP1- High exp

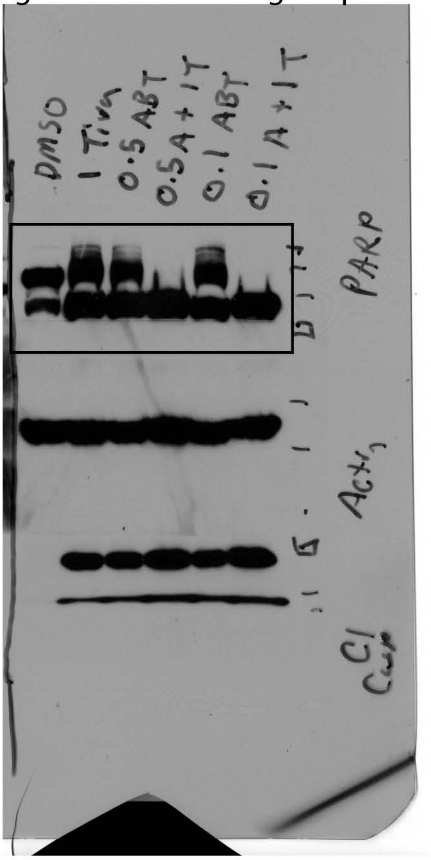

Figure 4c - PARP1- low exp (top)
Cl Casp 3 (bottom)

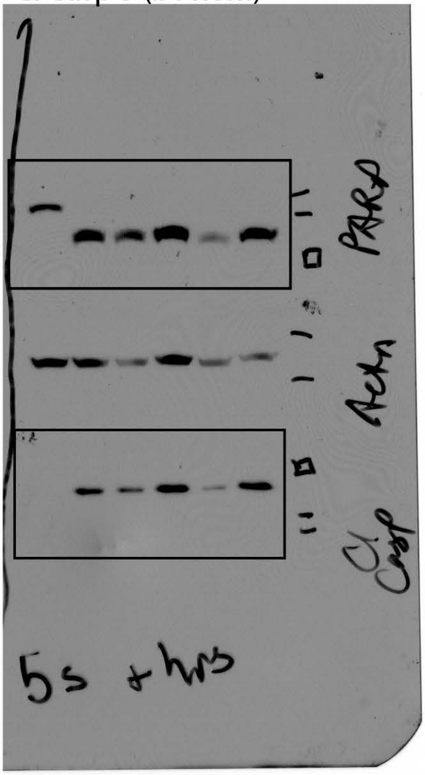

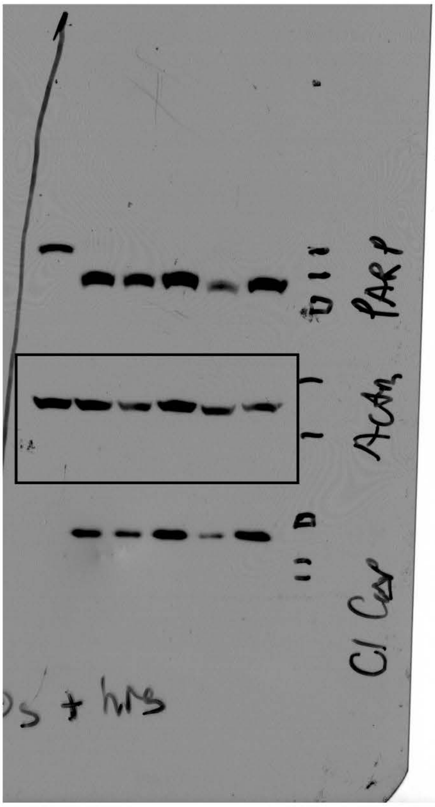

Figure 4c - actin

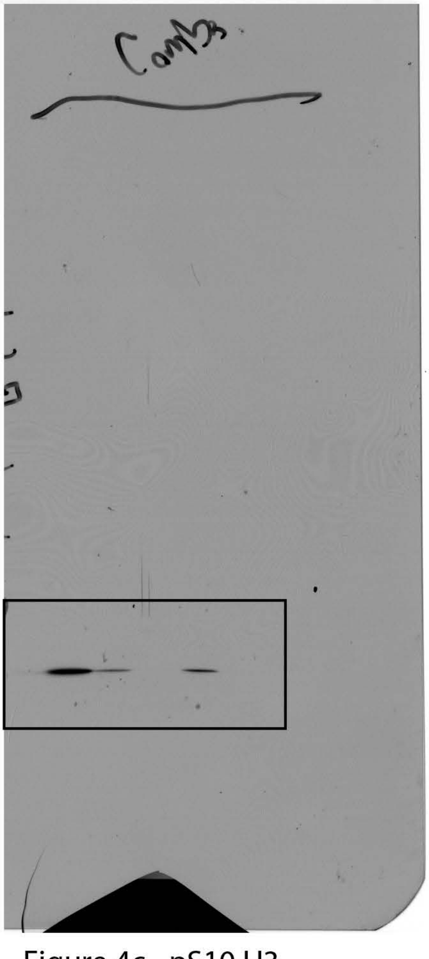

Figure 4c - pS10 H3

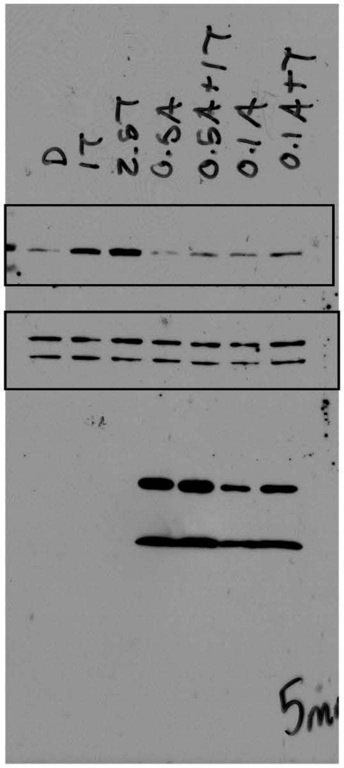

Figure 4d - 4h

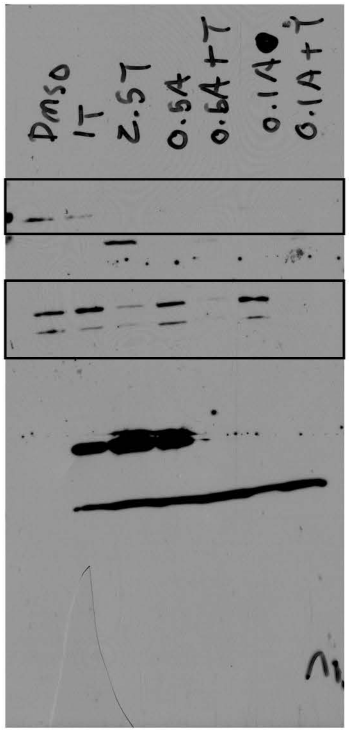

B-catenin

pY GSK3

Figure 4d - 24h

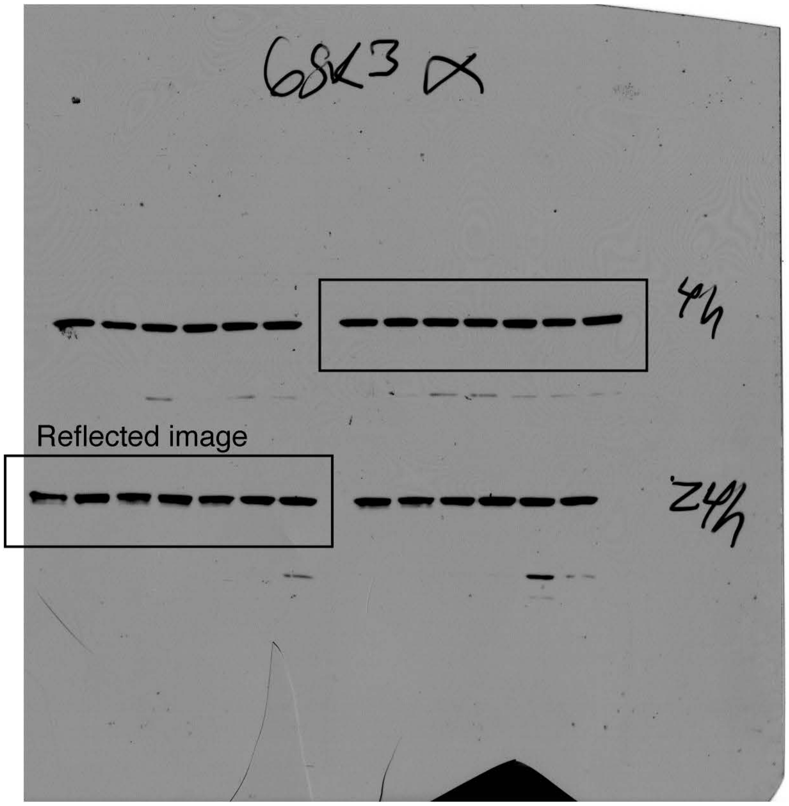

Figure 4d - 4h (top) - 24h (bottom) - GSK3a

** 24h GSK3a above is in reverse order (mirror image) of image in figure 4d **

Figure 4d - 24h actin

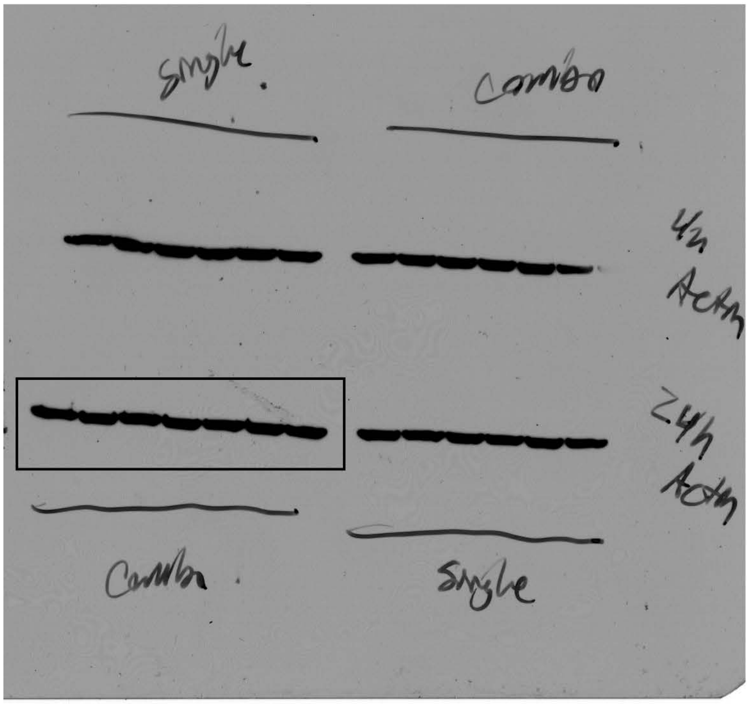

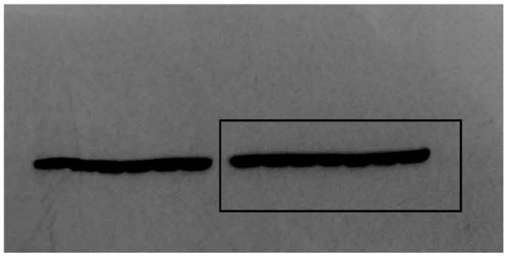

Figure 4d - 4h actin

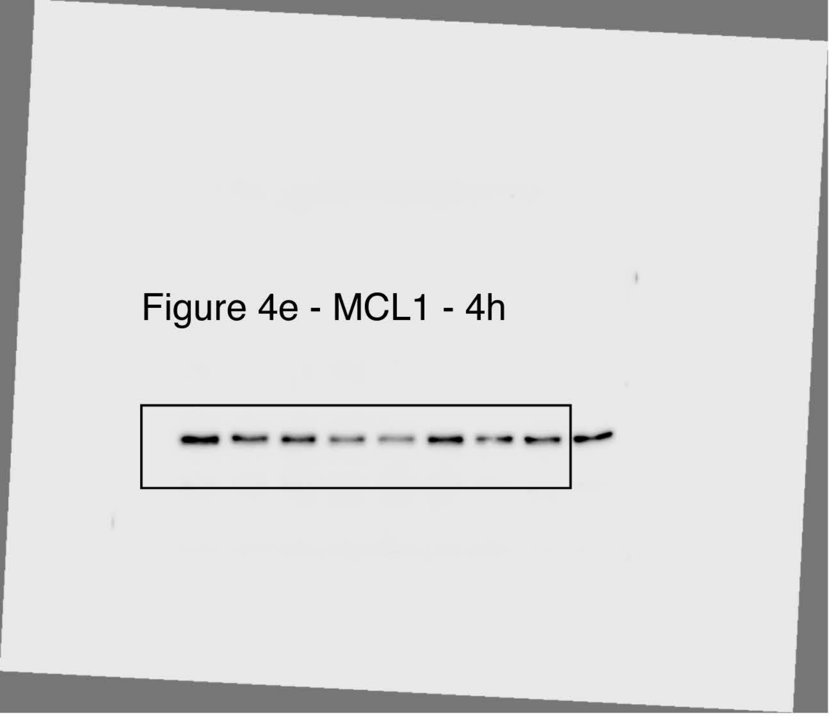

Figure 4e - Bak - 4h

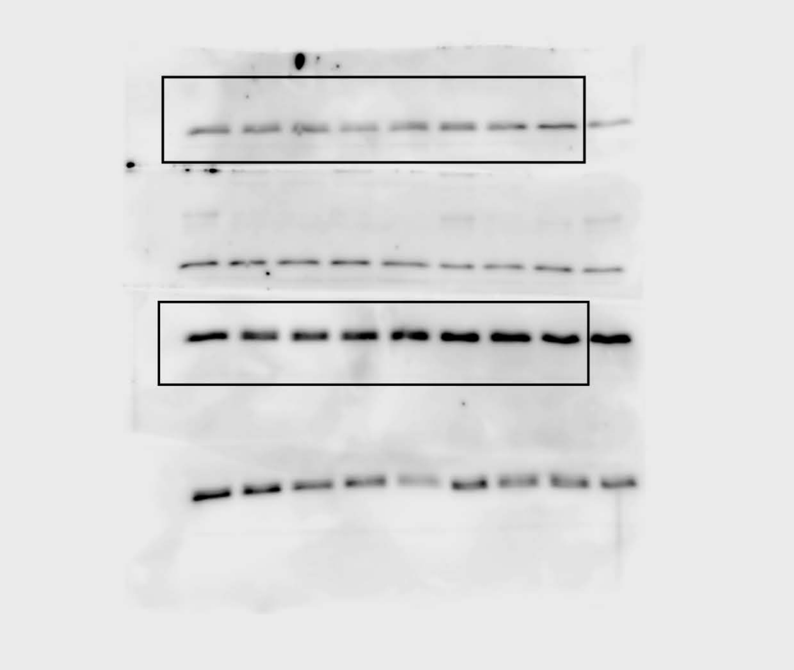

Figure 4e - BCLXL - 4h

Figure 4e - actin - 4h

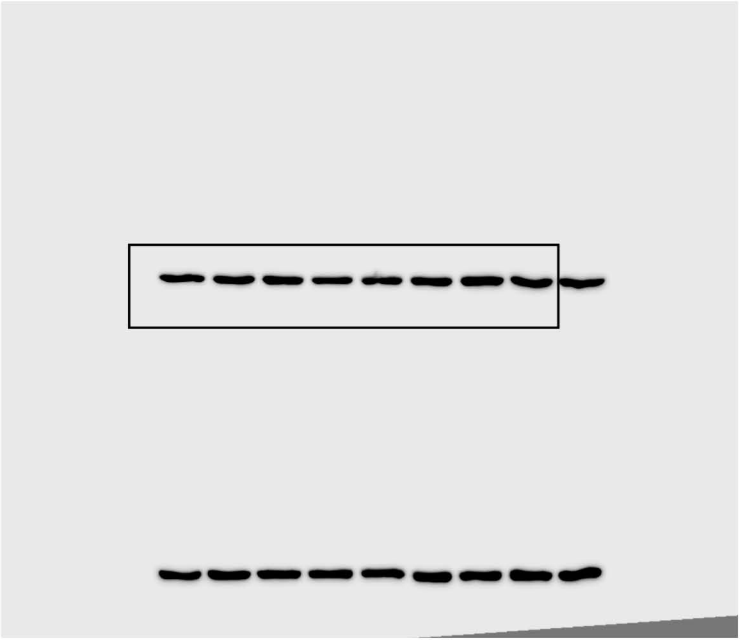

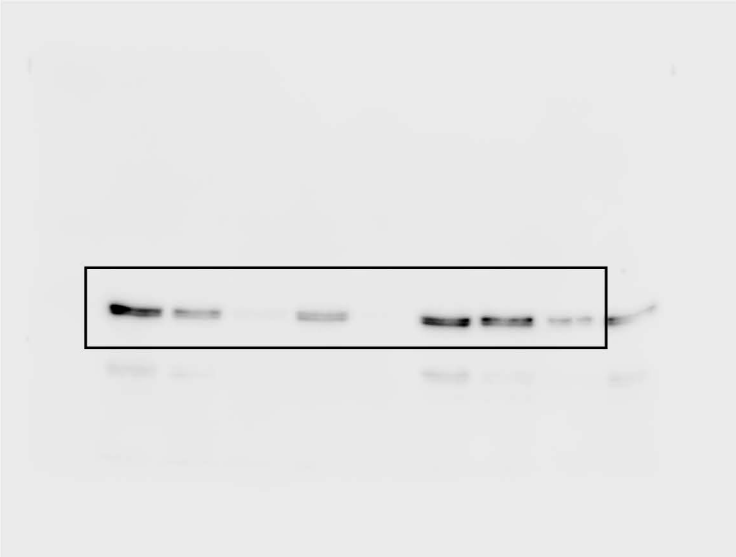

Figure 4e - MCL1 - 24h

Figure 4e - BCL-XL - 24h

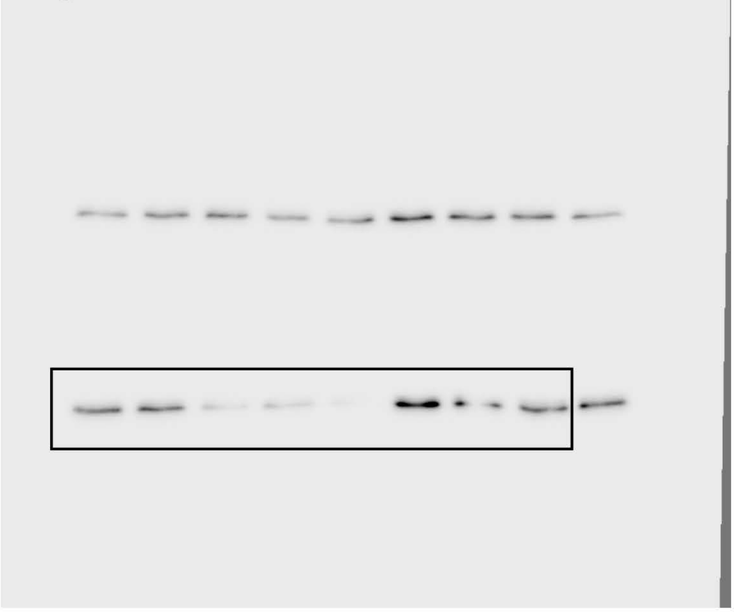

Figure 4e - Bak - 24h

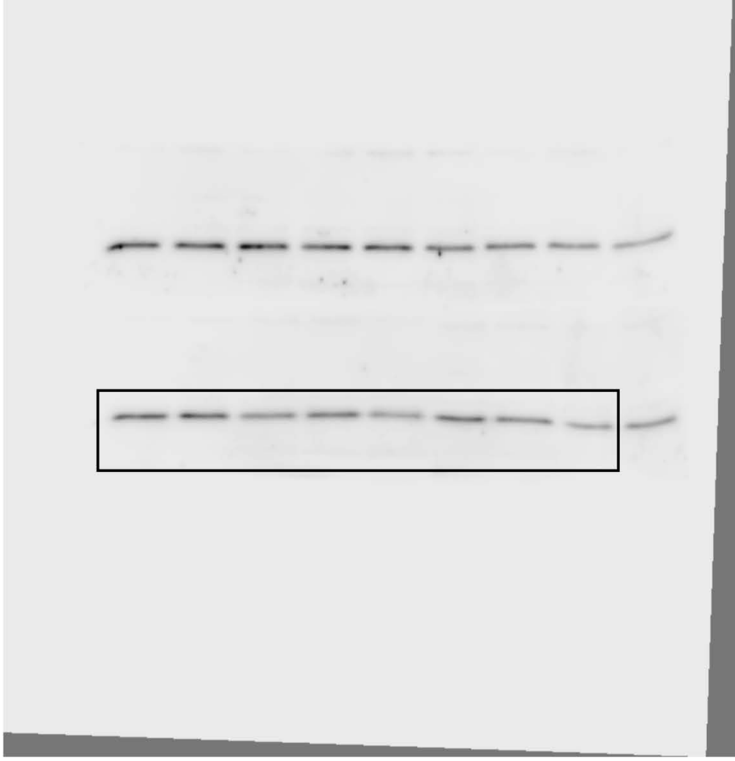

Figure 4e - 24h

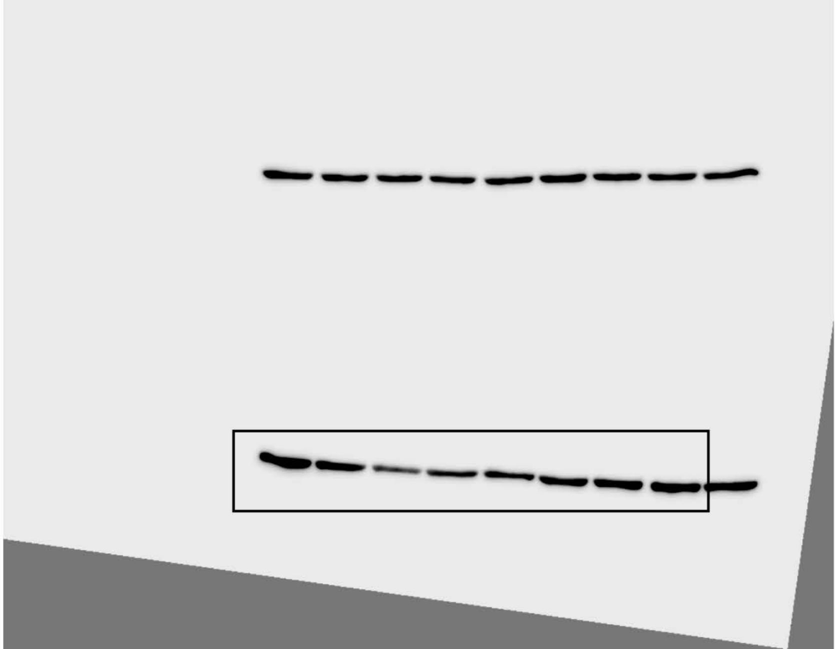

Supplement: Supplementary file 1 — Supplementary Information [file 41598_2018_37174_MOESM1_ESM.pdf]
